# Protocatechuate hydroxylase is a novel group A flavoprotein monooxygenase with a unique substrate recognition mechanism

**DOI:** 10.1016/j.jbc.2023.105508

**Published:** 2023-11-28

**Authors:** Nozomi Katsuki, Riku Fukushima, Yuki Doi, Shunsuke Masuo, Takatoshi Arakawa, Chihaya Yamada, Shinya Fushinobu, Naoki Takaya

**Affiliations:** 1 Faculty of Life and Environmental Sciences, Microbiology Research Center for Sustainability, University of Tsukuba, Tsukuba, Ibaraki, Japan; 2 Department of Biotechnology, The University of Tokyo, Tokyo, Japan; 3 Faculty of Pharmaceutical Sciences, Tokyo University of Science, Noda, Chiba, Japan; 4 School of Agriculture, Meiji University, Kawasaki, Kanagawa, Japan; 5 Collaborative Research Institute for Innovative Microbiology, The University of Tokyo, Tokyo, Japan

**Keywords:** enzyme kinetics, flavin adenine dinucleotide, *p*-hydroxybenzoate hydroxylase, pre-steady state kinetics, crystal structure, reductive half-reaction, pyridine nucleotide

## Abstract

*Para*-hydroxybenzoate hydroxylase (PHBH) is a group A flavoprotein monooxygenase that hydroxylates *p*-hydroxybenzoate to protocatechuate (PCA). Despite intensive studies of *Pseudomonas aeruginosa p*-hydroxybenzoate hydroxylase (PaPobA), the catalytic reactions of extremely diverse putative PHBH isozymes remain unresolved. We analyzed the phylogenetic relationships of known and predicted PHBHs and identified eight divergent clades. Clade F contains a protein that lacks the critical amino acid residues required for PaPobA to generate PHBH activity. Among proteins in this clade, *Xylophilus ampelinus* PobA (XaPobA) preferred PCA as a substrate and is the first known natural PCA 5-hydroxylase (PCAH). Crystal structures and kinetic properties revealed similar mechanisms of substrate carboxy group recognition between XaPobA and PaPobA. The unique Ile75, Met72, Val199, Trp201, and Phe385 residues of XaPobA form the bottom of a hydrophobic cavity with a shape that complements the 3-and 4-hydroxy groups of PCA and its binding site configuration. An interaction between the δ-sulfur atom of Met210 and the aromatic ring of PCA is likely to stabilize XaPobA-PCA complexes. The 4-hydroxy group of PCA forms a hydrogen bond with the main chain carbonyl of Thr294. These modes of binding constitute a novel substrate recognition mechanism that PaPobA lacks. This mechanism characterizes XaPobA and sheds light on the diversity of catalytic mechanisms of PobA-type PHBHs and group A flavoprotein monooxygenases.

Flavoprotein monooxygenases (FPMOs) are enzymes that transfer molecular oxygen (O_2_) atoms to various substrates *via* a prosthetic flavin adenine dinucleotide (FAD) or flavin mononucleotide (FMN) group. Organisms from bacteria to humans, produce FPMOs for detoxification, biosynthesis, and biodegradation (1). Eight FPMO groups have been classified based on amino acid sequence, tertiary structures, and cofactor preference (2). The largest group comprises FPMO A, which hydroxylates aromatic compounds using FAD as a prosthetic group. The co-substrates NADPH and NADH (NAD(P)H) reduce FAD to activate O_2_ (Fig. 1).

**Figure 1:**
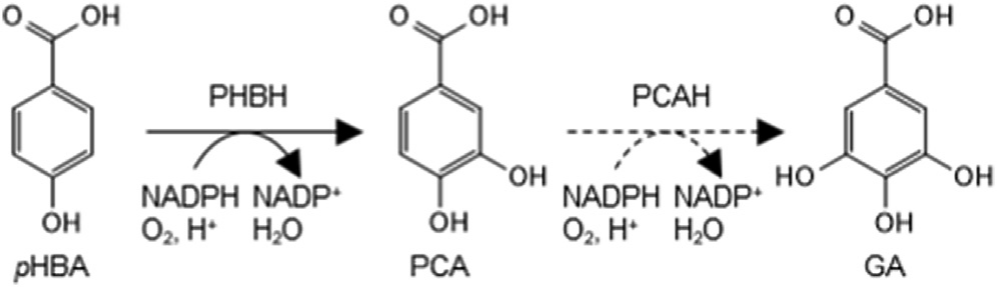
**Reactions of PHBH and PCAH**.

*Para*-hydroxybenzoate (*p*HBA) hydroxylase (PHBH) (E.C. 1.14.13.2) is a group A FPMO that hydroxylates *p*HBA to protocatechuate (PCA). The PobAs encoded by the *Pseudomonas aeruginosa* and *Pseudomonas fluorescens pobA* genes have 99.5% amino acid sequence identity and are excellent models of PHBH (3). The crystal structure of PobA from *P. fluorescens* (PfPobA) was initially determined in 1979 (4). Subsequent biochemical and structural studies then found that the PobA from *P. aeruginosa* PAO1 (PaPobA) has unique properties; *p*HBA bound to the enzyme moves the isoalloxazine ring of FAD and enhances FAD reduction by NADPH (5). Thereafter, PobA was classified according to its preference for NAD(P)H, to NADPH-specific, NAD(P)H-dependent, and NADH groups (6).

The catalytic cycle of PobA comprises reductive and oxidative half-reactions. The reductive half-reaction of PobA is initiated by the carboxy group of *p*HBA binding to the hydroxy groups of Ser212 and Tyr222 and the guanidino group of Arg214 according to the crystal structure of PaPobA (7, 8, 9) (Fig. S1*A*). The 4-hydroxy group of *p*HBA is hydrogen-bonded to the hydroxy group of Tyr201 and to carbonyl oxygen in the main chain of Pro293 (10). These amino acid residues are conserved among known PobAs and have been considered essential for activity. However, the novel enzymes revealed herein indicate otherwise. The 4-hydroxy group of *p*HBA constitutes a hydrogen-bond network with side chains of Tyr201, Tyr385, and His72, and two water molecules, and transfers protons between the catalytic center and the external solvent (11). This network lowers the p*K*_a_ of the 4-hydroxy group of *p*HBA by ∼2 units (12), resulting in electrostatic repulsion of the deprotonated 4-phenolate group with the carbonyl oxygen of Pro293, thus moving the isoalloxazine ring of FAD from an *inner* to an *outer* location (Fig. S1*B*) (10). This explains the 10^5^-fold higher FAD reduction rate in the presence of *p*HBA (13). After NADPH transfers a hydride anion, reduced FAD returns to the *inner* location. Subsequent oxidative half-reactions generate C4a-hydroperoxyflavin, then transfer an oxygen atom to the 3-position of *p*HBA.

Wild-type (WT) PaPobA specifically hydroxylates *p*HBA to protocatechuate (PCA) and the C5-carbon of PCA to generate gallic acid (GA) at a very low (∼1%) rate (14). However, GA is a value-added chemical (15, 16), so engineering PaPobA for PCA hydroxylation is important to several industries. An active site Y385F mutant has been created that hydroxylates PCA at a higher rate than WT PaPobA (14). Other mutations of T294A and L199V to Y385F have led to PCA hydroxylation rates that are 10- and 30-fold higher than that of WT PaPobA (17, 18). A PaPobA mutant with V47I/L199N/T294A/Y385I in the active site efficiently hydroxylates PCA (19). Structure modeling of the L199V/Y385F and the quadruple mutants predict a reorganized hydrogen-bond network involving the 3-hydroxy group of PCA that stabilizes bound PCA at a productive position (18, 19). All the above indicates that several mutant PobAs are involved in clarifying the structure-activity relationships of PobA.

A natural PobA that can efficiently hydroxylate PCA has remained unknown until now. We explored databases, identified evolutionally distant clades of PHBH family proteins, and detected a novel PCA 5-hydroxylase (PCAH) in *Xylophilus ampelinus* (XaPobA) and related bacteria. This enzyme shared substrate-binding Ser212, Arg214, Tyr222, and Pro293 residues with PaPobA and notably altered essential Tyr201 and Tyr385 during the PHBH activity of PaPobA to yield tryptophan and phenylalanine, respectively. The amino acid residues involved in binding *p*HBH and PCA largely differ between these PobAs. The XaPobA has a uniquely shaped hydrophobic cavity that is complementary to the substrate PCA. This stabilizes bound PCA, which stimulates FAD reduction by NAD(P)H. We propose a new mechanism of the PobA reaction based on these findings that should facilitate a rational design to alter the substrate specificity of this enzyme.

## Results

### Identifying novel groups of PobA

**Figure 2:**
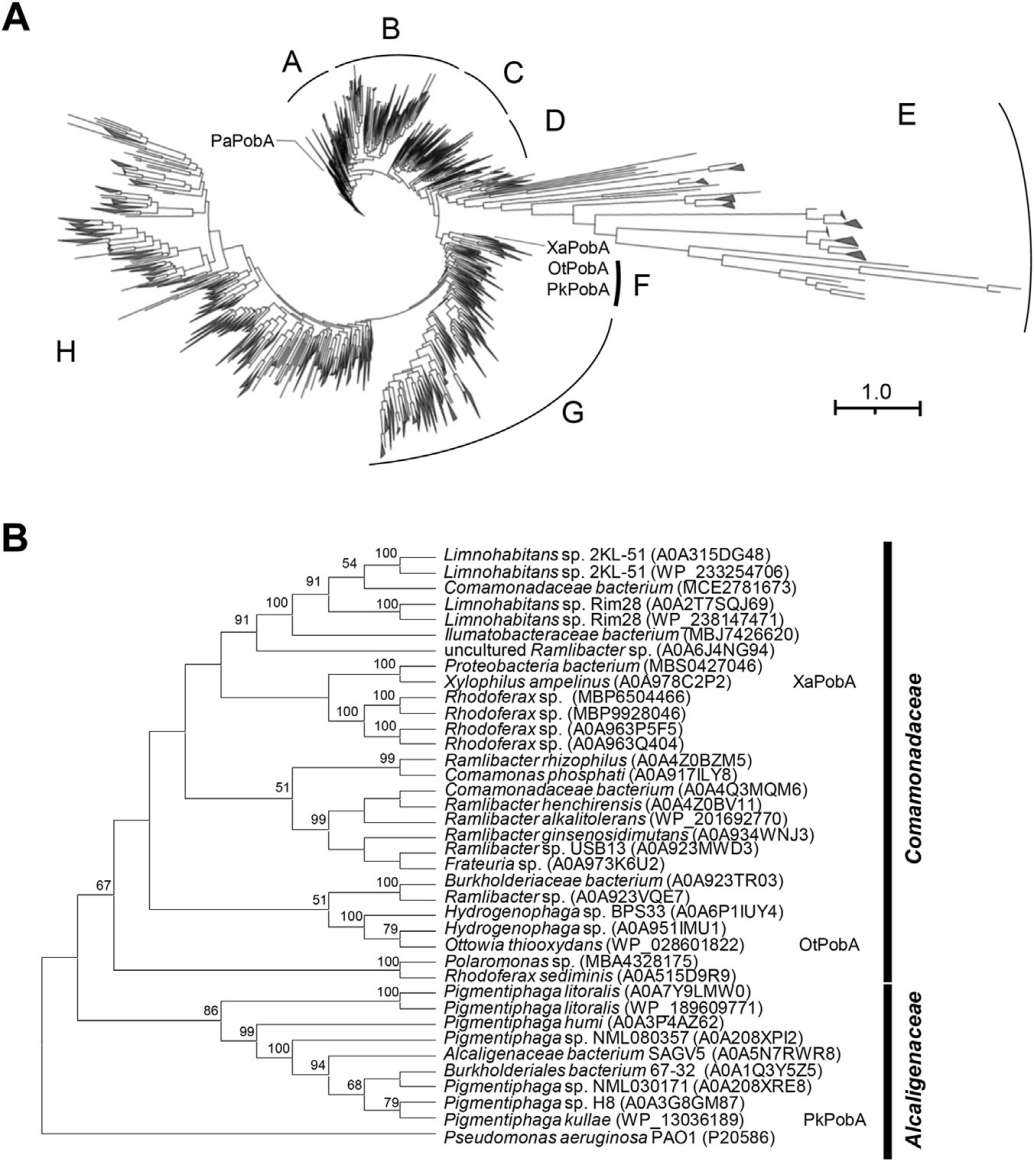
**Phylogenetic analysis of PobA proteins.***A*, putative PobAs. Amino acid sequences in UniProtKB/TrEMBL database were analyzed using MAFFT ver. 7 and FastTree 2.1. Proteins with an average branch length of <0.5 were collapsed into a single branch. *A*‒*H*, clades of putative PobAs; XaPobA, OtPobA, PkPobA, and PaPobA are located in the tree. *B*, phylogenetic analysis of clade F PobAs. Amino acid sequences were aligned using ClustalW, and their phylogeny was investigated using MEGA 11. Bootstrap values and branches were calculated using 1000 bootstrap resampling replicates. Accession numbers are shown in parentheses.

The amino acid sequences of predicted PobA proteins annotated as 4-hydroxybenzoate 3-monooxygenases in group A FPMOs were downloaded from the UniProtKB/TrEMBL databases. Phylogenetic analysis of 8127 sequences revealed eight clades (Figs. 2*A* and S2). Clade A contained the most studied PaPobAs and other NADPH-dependent PHBHs. Clades C and G contained PobA produced by *Cupriavidus necator* JMP134 and *Actinomyces* bacteria, respectively, with biochemical properties that have been characterized (6, 20). Clades B, D, E, F, and H have no PHBHs with biochemical properties characterized. Clade A, B‒D, and G‒H respectively correspond to NADPH-specific, NAD(P)H-dependent, and NADH-preferring groups classified as described (6). These findings indicated that the phylogeny is relevant to the NAD(P)H preference of PobAs. Clades E and F do not include a characterized PHBH except for the recently discovered PHBH isozyme (21). Clade E contains proteins from *Thermus-Deinococcus, Pseudomonadota, Actinomycetota*, and *Chloroflexi*. Long branches in this clade indicated their evolution within specific environments rather than bacterial lineage. We therefore focused on characterizing PobAs in clade F.

A search of the National Center for Biotechnology Information database (National Institutes of Health [NIH], Bethesda, MD, USA) for proteins with amino acid sequence similarity to those in clade F yielded 33 proteins. We merged them with the 24 proteins in clade F to create an expanded protein list after removing duplications. The resulting 37 proteins mostly originated from bacteria in the order *Burkholderiales* of the *Betaproteobacteria*. The amino acid sequence identities of these proteins to conventional PaPobA (clade A), PobA from *Acinetobacter baylyi* ADP1 (AbPobA, clade C) (22), and *Corynebacterium glutamicum* ATCC13032 (CgPobA, clade G) (20) were <47%, 41%, and 39%, respectively. Phylogenetic analysis showed that the clade F proteins clustered into two groups, each consisting of proteins from bacteria in the families *Alcaligenaceae* and *Comamonadaceae* (Fig. 2*B*). These findings suggested that clade F proteins diversified as these bacteria evolved.

### Clade F includes novel PCA hydroxylase (PCAH)

**Figure 3:**
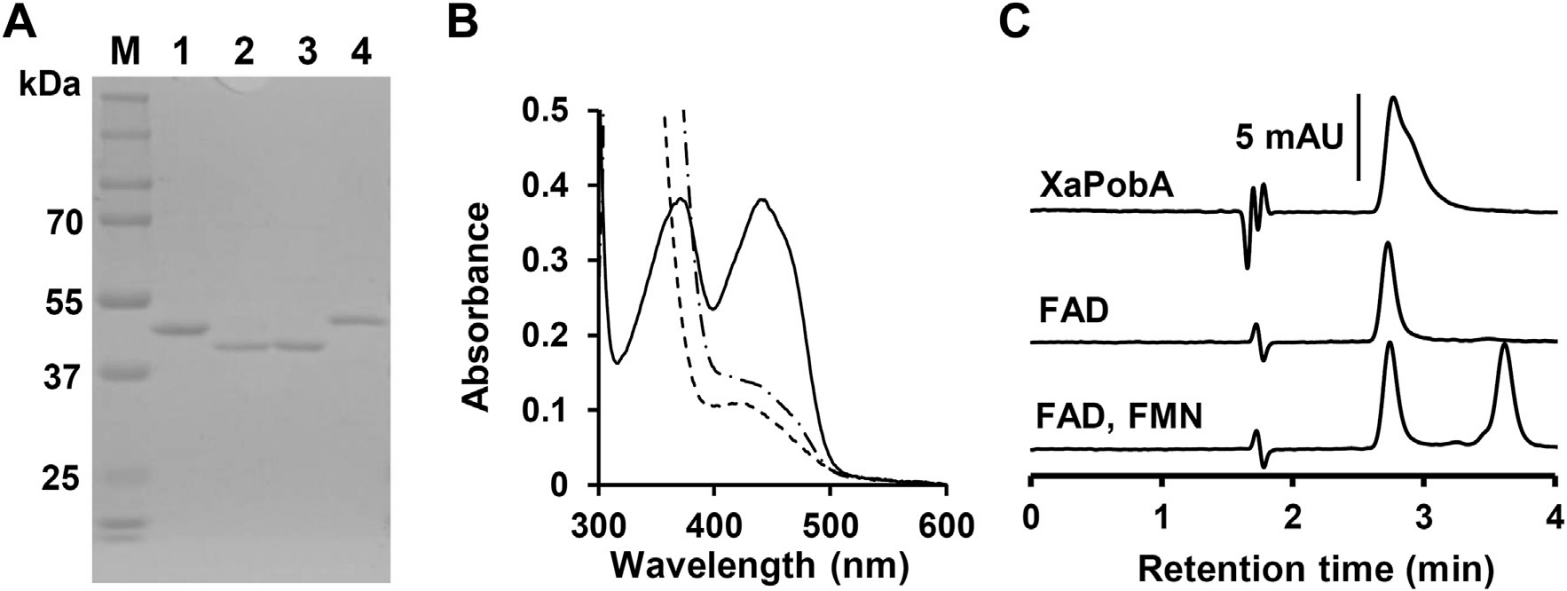
**Biochemical characterization of clade F PobAs.***A*, SDS-PAGE. Lanes: M, molecular mass marker; 1, XaPobA; 2, OtPobA; 3, PkPobA; 4, PaPobA (1 μg each). *B*, absorption spectra of XaPobA (57 μM) in 20 mM Tris-HCl (pH 7.9). Solid line, purified oxidized XaPobA; dashed line, XaPobA reduced by sodium hydrosulfite; dash-dotted line, XaPobA reduced by 0.18 mM NADPH in 2 mM PCA under anaerobic conditions. *C*, analysis of FAD in XaPobA. Heated extract of XaPobA along with commercial FAD and FMN were analyzed using HPLC.

We prepared recombinant proteins from the predicted clade F PobAs in *X. ampelinus* CCH5-B3 (XaPobA, accession number A0A978C2P2), *Ottowia thiooxydans* DSM 14619 (OtPobA, WP_028601822), and *Pigmentiphaga kullae* DSM 13708 (PkPobA, WP_130361895). We confirmed that purity and molecular masses of the recombinant and calculated proteins were similar using SDS-PAGE (Fig. 3*A*). The visible absorption spectrum of these PobAs had peaked at 375 and 440 nm (Figs. 3*B* and S3) that were decreased by sodium hydrosulfite and by NADPH plus PCA under anaerobic conditions. The findings of high-performance liquid chromatography (HPLC) showed that the clade F PobAs contained FAD but not FMN (Figs. 3*C* and S3), indicating that XaPobA, OtPobA, and PkPobA use FAD as a prosthetic group.

The initial velocities of PCA-dependent NADPH oxidation by XaPobA, OtPobA, and PkPobA were 1.8 ± 0.2 × 10^3^, 2.7 ± 0.2 × 10^3^, and 1.7 ± 0.3 × 10^3^ nmol min^−1^ mg^−1^ (Table S1). Those dependent on *p*HBA were 4.8 ± 0.7 × 10^1^, 8.7 ± 1.8 × 10^2^, and 1.1 ± 0.4 × 10^2^ nmol min^−1^ mg^−1^, respectively, indicating that XaPobA, OtPobA, and PkPobA preferred PCA to *p*HBA. Replacing NADPH with NADH reduced the activity to 0.7%, 0.9%, and 2% of that of NADPH (Table S1), indicating that these PobA proteins prefer NADPH as a reductant. Analyzing PCA and NADPH reactions by HPLC identified GA (Fig. S4). These results indicated that the clade F PobAs are NADPH-dependent PCA hydroxylases (PCAHs) that generate GA as a reaction product.

### Biochemical characteristics of XaPobA

We investigated the catalytic properties of XaPobA in detail. Besides PCA and *p*HBA, activity for *p*-aminobenzoic acid, hydroquinone, catechol, *o*-hydroxybenzoic, *m*-hydroxybenzoic, 2,4-dihydroxybenzoic, *p*-coumaric, and caffeic acid was below the limits of detection (Table S1), indicating that the XaPobA reaction is specific. The initial velocities of PCA/*p*HBA-dependent NADPH oxidation were fitted to the Michaelis-Menten equation (Fig. S5). The apparent Michaelis (*K*_m_) and rate (*k*_cat_) constants for PCA were 3.7 ± 0.9 × 10^−1^ mM and 1.8 ± 0.1 × 10^0^ s^−1^, respectively, and the *k*_cat_ value was 38-fold higher than that for *p*HBA (4.7 ± 0.4 × 10^−2^ s^−1^) (Table 1). The *k*_cat_ value for PCA of the XaPobA was 3.5-fold higher than that of the typical PHBH, PaPobA. The *k*_cat_ value for *p*HBA was only 1% of that of PaPobA. Initial velocities of the reaction did not saturate up to 0.25 mM NAD(P)H and *K*_m_ and *k*_cat_ values for NAD(P)H were not determined (Fig. S5). The coupling ratio of NADPH consumption *versus* GA or PCA production (mol/mol) by the reaction with *p*HBA was 18%, implying that a significant portion of NADPH in the reaction was wasted to reduce O_2_ (Table 1). The coupling ratio in the reaction with PCA was 53%, which was 3-fold higher than that with *p*HBA. These results indicated that XaPobA prefers PCA over *p*HBA as a substrate. The preference for and the coupling reaction with PCA were better for PkPobA and OtPobA (Table 1).

**Table 1:** Apparent steady-state kinetic parameters of XaPobA, OtPobA, PkPobA, and PaPobA

Enzyme	PCA				*p*HBA			
	*k*_cat_ (s^−1^)	*K*_m_ (mM)	*k*_cat_*/K*_m_ (mM^−1^ s^−1^)	Coupling ratio (%)	*k*_cat_ (s^−1^)	*K*_m_ (mM)	*k*_cat_*/K*_m_ (mM^−1^ s^−1^)	Coupling ratio (%)
XaPobA	1.8 ± 0.1 × 10^0^	3.7 ± 0.9 × 10^−1^	4.9 ± 1.1	53	4.7 ± 0.4 × 10^−2^	4.3 ± 0.3 × 10^−1^	1.1 ± 0.3 × 10^−^^1^	18
OtPobA	3.5 ± 0.3 × 10^0^	5.3 ± 1.9 × 10^−1^	7.6 ± 2.8	36	6.3 ± 1.9 × 10^−1^	7.7 ± 2.6 × 10^−2^	8.2 ± 0.4 × 10^0^	22
PkPobA	1.1 ± 0.1 × 10^0^	3.2 ± 0.7 × 10^−1^	3.7 ± 0.6	36	1.1 ± 0.3 × 10^−1^	3.7 ± 1.8 × 10^−1^	3.4 ± 0.9 × 10^−1^	9
PaPobA	5.1 ± 0.2 × 10^−1^	1.3 ± 0.2 × 10^−1^	4.1 ± 0.9	3	4.7 ± 0.3 × 10^0^	2.4 ± 0.7 × 10^−2^	2.0 ± 0.4 × 10^2^	77

Initial velocity of NADPH consumption was measured in 20 mM Tris-HCl (pH 7.9) containing 0.25 mM NADPH, 0 to 2 mM PCA or *p*HBA, and purified PobAs at 25 °C. Data were means ± standard deviations of triplicate results.

**Figure 4:**
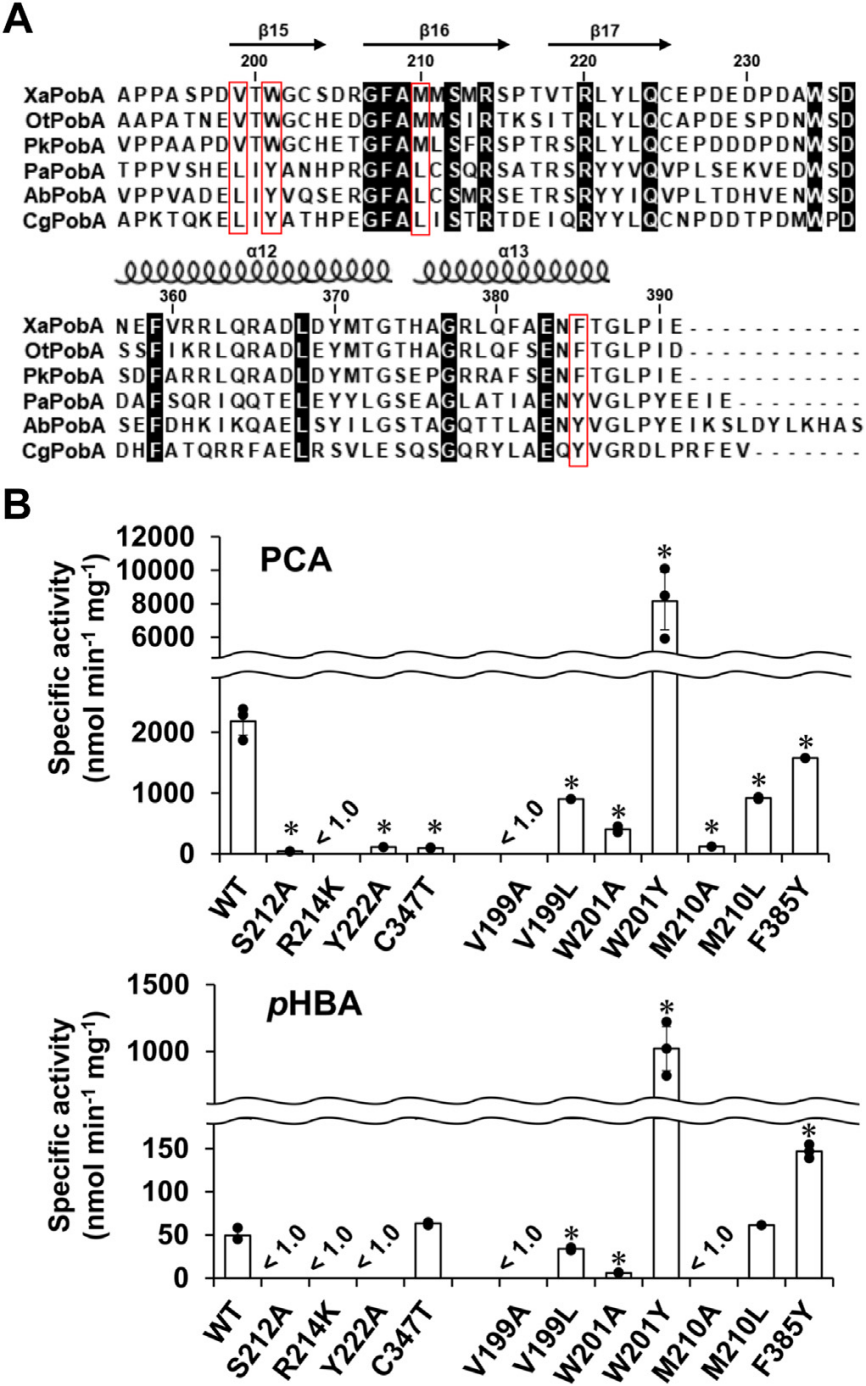
**Multiple sequence alignment of PobA proteins and role of conserved residues in enzyme activity.***A*, partial amino acid sequences of XaPobA, OtPobA, and PkPobA along with PobA in the other clades (PaPobA, AbPobA, and CgPobA) were aligned using Clustal W. Secondary structures are according to XaPobA (PDB ID: 8JQP). *Black boxes* indicate identical residues among sequences. Characteristic amino acids among clade F PobAs are boxed in red. *B*, specific activity of XaPobA and its mutants for PCA (*upper panel*) and *p*HBA (*bottom panel*). The initial velocity of NADPH oxidation was determined by measuring absorption at 340 nm. Data are means ± standard deviation, *n* = 3. ∗*p* < 0.05 (WT *versus* mutant, unpaired Student’s *t* test).

We compared whole amino acid sequences of XaPobA, OtPobA, and PkPobA in clade F with typical PobAs in other clades (Figs. 4*A* and S6). The known catalytically important amino acid residues (Leu199, Tyr201, Leu210, and Tyr385) in the catalytic site of PaPobA were respectively replaced in Clade F PobAs with Val199, Trp201, Met210, and Phe385. These replacements were common to 37 clade F proteins (Fig. 2*B*) except Ile199 was substituted with Val199 in nine predicted PobAs (Accession numbers, MBP6504466, MBL8382957, WP_233254706, WP_201692770, WP_238147471, MCE2781673, MBJ7426620, MBA4328175, A0A934WNJ3). We investigated the roles of these residues in alanine-substituted mutants of XaPobA. However, we could not produce an F385A mutant. The specific activity of the V199A, W201A, and M210A mutants against PCA was lower than that of WT XaPobA, and that against *p*HBA was almost eliminated (Fig. 4*B*), indicating the importance of Val199, Trp201, and Met210 for substrate specificity. The side-chain hydroxy group of Tyr201 is essential for PaPobA to bind the *p*HBA substrate (13). However, this residue is substituted in XaPobA with Trp201 which lacks a side chain hydroxy group. This indicated that XaPobA might recognize substrates *via* a novel mechanism. The Ser212, Arg214, and Tyr222 residues, which bind the carboxy group of *p*HBA in PaPobA, were conserved in clade F and other PobAs (Fig. 4*A*). The S212A, R214K, and Y222A (R214A protein could not be prepared due to insolubility) mutants of XaPobA generated little activity against PCA and *p*HBA (Fig. 4*B*). The function of these residues to stabilize the enzyme-substrate complex seems to be common to PaPobA and XaPobA.

### Crystal structure of XaPobA

**Figure 5:**
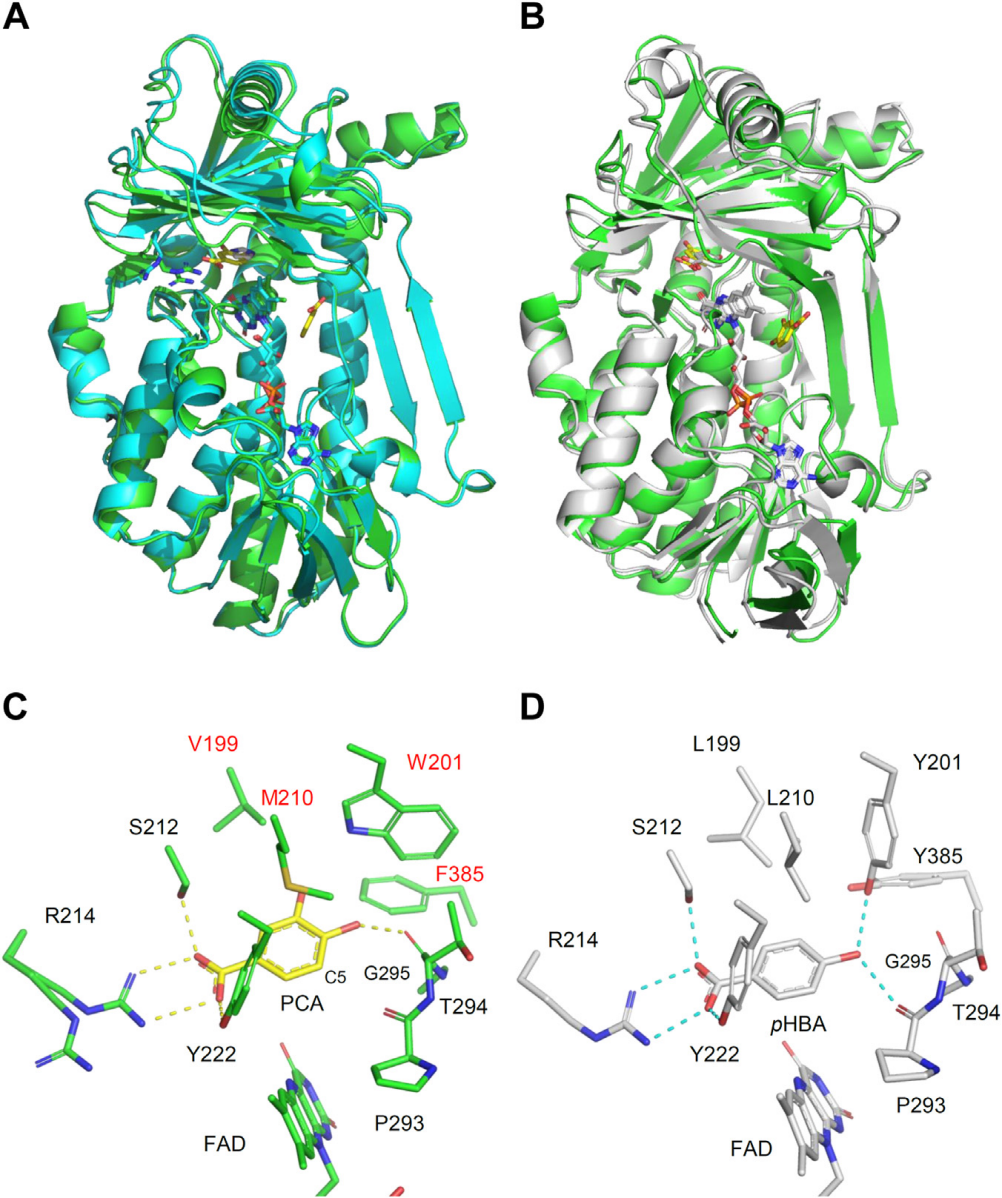
**Crystal structure of XaPobA.***A*, superimposed XaPobA + imidazole (*cyan*) and XaPobA + PCA (*green*). FAD (*cyan or green sticks*), imidazole (*white sticks*), and PCA (*yellow sticks*). *B*, superimposed XaPobA + PCA (*green*) and PaPobA + *p*HBA (PDB ID: 1IUW, *white*). *C*, active site of XaPobA + PCA. *Yellow dashed lines*, hydrogen-bonded PCA. Red letters and numbers, characteristic amino acids in clade F PobA proteins. *D*, active site of PaPobA + *p*HBA. *Dashed cyan lines*, hydrogen bonding to *p*HBA.

We studied the crystallographic structure of XaPobA to gain structural insight into the unique substrate specificity. We initially determined the crystal structure of XaPobA at a resolution of 1.6 Å (Table S2). The overall structure of XaPobA comprised a homodimer, with each unit binding an oxidized form of FAD in its active site (Fig. S7*A*). The XaPobA monomer consisted of FAD-(amino acids 1–73, 88–198, and 267–391)- and substrate (amino acids 74–87 and 199–266)- binding domains (Fig. 5*A*). An imidazole molecule derived from the crystallization buffer bound to the active site of this structure (XaPobA + imidazole) (Fig. S7*B*). The imidazole molecule was surrounded by hydrophobic side chains of Val199, Met210, Trp201, and Phe385, and a hydrogen bond was formed with the main chain amide of Thr294. The Arg214 side chain, which anchors the carboxy group of the substrate *p*HBA in PaPobA (Fig. S1), swung out and was not in a conformation suitable for binding to the canonical substrate. To understand the substrate recognition mechanism of XaPobA, we determined the PCA-bound structure (XaPobA + PCA) at a resolution of 1.65 Å by growing crystals under imidazole-free conditions and soaking them with PCA (Table S2). The β-sheets 14 to 16 in the substrate binding domain (Figs. 4*A* and S6) were located significantly closer to the catalytic center in XaPobA + PCA compared with XaPobA + imidazole (Fig. 5*A*), indicating that bound PCA induced XaPobA to adopt a closed conformation (18, 23). Hereinafter, we mainly describe the XaPobA + PCA structure. The structures of the main chains in XaPobA and PaPobA (PDB ID: 1IUW) were very similar, and superimposed with a Cα root mean square deviation (RMSD) of 0.86 Å (Fig. 5*B*). These structures contained an FAD molecule oriented at the *inner* position (Fig. S8*A*), which corresponded to that during PCA oxygenation. The flavin orientation closely agreed with the *p*HBA-bound PaPobA (Fig. S8*B*), but not at the *outer* position in PaPobA R222Q (PDB ID: 1K0I) (Fig. S8, *C* and *D*) (23).

### PCA binding site of XaPobA

The PCA molecule in the active site was adjacent to the isoalloxazine ring of FAD with the aromatic C5 atom to be hydroxylated facing the ring (Fig. 5*C*). The binding orientation of the PaPobA Y385F mutant that can hydroxylate PCA (Fig. S9*A*) was similar (18). This was in contrast to PfPobA, which bound PCA with the hydroxylated C3 atom facing FAD in an inert orientation and produced little PCAH activity (Fig. S9*B*) (24). These results indicated that the binding orientation of PCA in the crystal structures correlated to the level of PCAH activity of PobAs, and is catalytically productive. The carboxy group of PCA in the XaPobA + PCA structure hydrogen-bonded with the side chain hydroxy groups of Ser212 and Tyr222 (Fig. 5*C*). The side chain of Arg214 had two alternative conformations, one of which formed a salt bridge with the carboxy group of PCA. Therefore, these residues were essential for stabilizing PCA binding. This was consistent with the finding that XaPobA with mutant Ser212 and Tyr222 almost completely lost PCAH and PHBH activities (Fig. 4*B*). These amino acid residues were also involved in binding between PaPobA and *p*HBA (Fig. 5*D*) (7‒9), suggesting that XaPobA and PaPobA have the same binding mechanisms.

In addition to PCA in the active site, we also identified the electron density of an apparently catalytically inert second PCA molecule (Fig. S7*C*) located near the ribityl moiety of FAD at ∼6 Å from the isoalloxazine ring (Fig. S8*A*). A second substrate binding site was also found in PaPobA R220Q soaked in high concentrations of *p*HBA as a low-affinity site (Fig. S8*C*) (22). Superimposing the XaPobA and PaPobA structures showed that the positions of the bound PCA and *p*HBA partly overlapped (Fig. S8*D*). The low-affinity site might be an entry route of *p*HBA to the active site of XaPobA (22). However, further investigation is needed to verify this.

### Trp201, Val199, and Met210 in PCA-dependent FAD reduction

The XaPobA + PCA structure revealed unique residues located near the catalytic center. Side chains of Val199 and Met210 surrounded PCA without hydrogen bonding (Fig. 5*C*). The Trp201 and Phe385 residues of XaPobA occupied the distal region of the catalytic site and were too distant to form hydrogen bonds with PCA. This was in contrast to PaPobA, in which hydroxy groups of the corresponding Tyr201 and Tyr385 were hydrogen bonded to *p*HBA (Fig. 5*D*). We focused on the unique Val199, Trp201, and Met210 residues involved in the PCAH activity (Fig. 4*B*). The V199A mutant caused almost a total loss of activity towards PCA and *p*HBA (*k*_cat_ < 1 × 10^−3^, Table 2). Steady-state kinetics of PCA-dependent NADPH oxidation by the W201A and M210A mutants determined *k*_cat_ values of 9.3 ± 1.1 × 10^−2^ and 1.6 ± 0.1 × 10^−1^ s^−1^, respectively, which were 19- and 11-fold lower than WT XaPobA, whereas the *K*_m_ values increased 1.8- and 2.6-fold. Both mutations had decreased coupling ratios of NADPH oxidation and GA production compared with WT XaPobA (Table 2). These results indicated that the Trp201 and Met210 residues are involved in catalytic turnover by determining the geometry of the bound substrates.

**Table 2:** Apparent steady-state kinetic parameters of XaPobA and its mutants

Enzyme	Substrate	*k*_cat_ (s^−1^)^a^	*K*_m_ (mM)	*k*_cat_*/K*_m_ (mM^−1^ s^−1^)	*k*_red_ (s^−1^)^b^	Coupling ratio (%)
WT	PCA	1.8 ± 0.1 × 10^0^	3.7 ± 0.9 × 10^−1^	4.9 ± 1.1 × 10^0^	2.0 ± 0.0 × 10^1^	53
	*p*HBA	4.7 ± 0.4 × 10^−2^	4.3 ± 0.3 × 10^−1^	1.1 ± 0.3 × 10^−1^	8.1 ± 0.1 × 10^−1^	18
V199A	PCA	<1 × 10^−3^	N. A.^c^	N. A.	<1 × 10^−3^	N. A.
	*p*HBA	<1 × 10^−3^	N. A.	N. A.	<1 × 10^−3^	N. A.
W201A	PCA	9.3 ± 1.1 × 10^−2^	6.5 ± 2.7 × 10^−1^	1.4 ± 0.4 × 10^−1^	1.6 ± 0.0 × 10^−1^	35
	*p*HBA	<1 × 10^−3^	N. A.	N. A.	1.2 ± 0.1 × 10^−1^	N. A.
W201Y	PCA	8.1 ± 0.6 × 10^0^	5.0 ± 1.1 × 10^−1^	1.6 ± 0.3 × 10^1^	4.0 ± 0.1 × 10^1^	46
	*p*HBA	7.9 ± 0.7 × 10^−1^	7.2 ± 2.2 × 10^−2^	1.1 ± 0.3 × 10^1^	4.4 ± 0.0 × 10^1^	42
M210A	PCA	1.6 ± 0.1 × 10^−1^	9.6 ± 0.3 × 10^−1^	1.6 ± 0.4 × 10^−^^1^	9.9 ± 0.1 × 10^0^	2
	*p*HBA	<1 × 10^−3^	N. A.	N. A.	5.5 ± 0.3 × 10^−1^	N. A.
C347T	PCA	8.3 ± 0.4 × 10^−2^	3.2 ± 1.0 × 10^−1^	2.9 ± 0.9 × 10^−1^	6.5 ± 0.7 × 10^−1^	19
	*p*HBA	8.6 ± 1.6 × 10^−2^	9.1 ± 3.4 × 10^−1^	1.0 ± 0.3 × 10^−^^1^	4.0 ± 0.1 × 10^−1^	0.4

a Initial velocity of NADPH consumption was measured in 20 mM Tris-HCl (pH 7.9) containing 0.25 mM NADPH, 0 to 2 mM PCA or *p*HBA, and purified PobAs at 25 °C. Data were means ± standard deviations (*n* = 3).

b Pre-steady state reaction was measured in 20 mM Tris-HCl (pH 7.9) containing 8 mM NADPH, 2 mM PCA or *p*HBA, 2 mM D-glucose, 10 units mL^−1^ glucose oxidase, 150 units mL^−1^ catalase, and purified PobAs at 25 °C under the anaerobic conditions. Data were means ± standard deviations (*n* = 3).

c N. A., not applicable.

**Figure 6:**
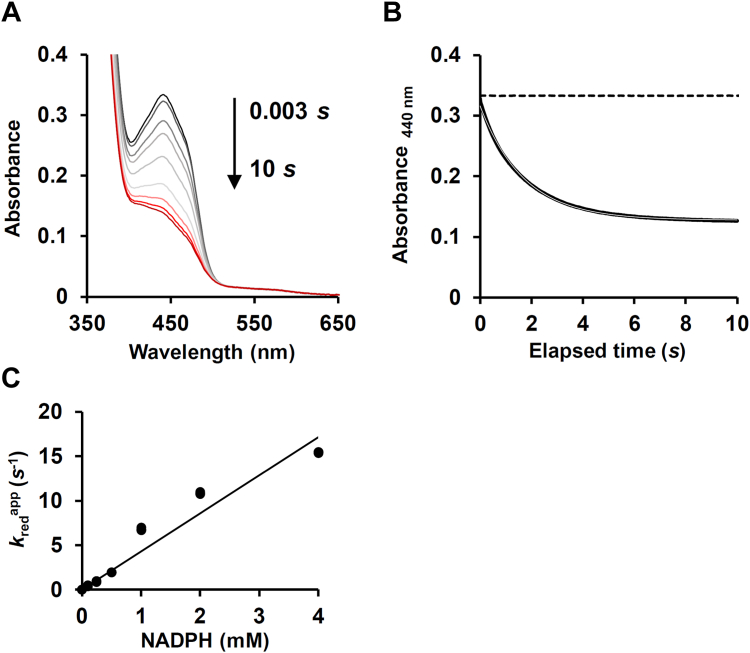
**Pre-steady state reduction of XaPobA.***A*, visible absorption spectra of XaPobA measured using stopped-flow rapid-scan spectrophotometry. Enzyme solution (30 μM XaPobA, 2 mM PCA in 20 mM Tris-HCl, pH 7.9) and 0.18 mM NADPH were rapidly mixed at 25 °C under anaerobic conditions, and 5000 spectra were collected over 10 s. Selected spectra are shown for clarity. *B*, PCA-dependent FAD reduction. Absorbance at 440 nm changed by reactions in *Panel A* (*solid line*), the single exponential fitting (*solid gray line*), and without PCA (*dotted line*). (*C*) NADPH dependence of FAD reduction. Triplicate reactions were measured as shown in *panel A* using 1 to 4 mM NADPH.

We assessed the roles of Val199, Trp201, and Met210 residues on the reductive half-reaction using pre-steady state kinetics and stopped-flow rapid-scan spectrophotometry. An absorption peak of oxidized XaPobA at 440 nm decreased when XaPobA, excess NADPH, and PCA were mixed under anaerobic conditions (Fig. 6*A*). The spectrum at 10 s after mixing resembled that of XaPobA equilibrated with NADPH in the presence of PCA (Fig. 3*B*). A rapid reaction followed pseudo-first-order kinetics with apparent rate constants (*k*_red_) of 2.0 ± 0.0 × 10^1^ s^−1^ in the presence of PCA, and 8.2 ± 0.1 × 10^−1^ s^−1^ in the presence of *p*HBA (2 mM each) (Fig. 6*B*, Table 2). The faster reaction rate in the presence of PCA coincided with the PCA preference for XaPobA. Little FAD was reduced in the absence of PCA and *p*HBA (*k*_red_ < 1.0 × 10^−4^ s^−1^), indicating that PCA/*p*HBA must bind to XaPobA before FAD can be reduced as in PaPobA (13). Increasing the concentration of NADPH to 4 mM did not saturate the FAD reduction rate, and the biomolecular rate constant was 4.0 ± 0.0 × 10^0^ mM^−1^ s^−1^ (Fig. 6*C*). The *k*_red_ values of V199A, W201A, and M210A mutant reactions in the presence of PCA were < 1.0 × 10^−4^, 1.6 ± 0.0 × 10^−1^, and 9.9 ± 0.1 × 10^0^ s^−1^, respectively, and >10^4^-, 120-, and 2-fold lower than the *k*_red_ value of WT XaPobA (Table 2). These results were in line with the notion that these residues bind PCA.

We analyzed XaPobA mutants with V199L, W201Y, M210L, and F385Y substitutions that mimic PaPobA to understand the functional relevance of these residues and the preference for PCA/*p*HBA (Fig. 4*B*). The V199L and M210L mutations decreased PCAH, while maintaining PHBH activity, indicating that these residues are responsible for recognizing PCA, but not *p*HBA. Substituting these residues with alanine largely decreased both activities (Fig. 4*B*), indicating that the hydrophobic side chains of the residues determine the PCA/*p*HBA preference. The F385Y mutant generated less PCAH and more PHBH activities than WT XaPobA; thus, the F385Y residue switches the PCA and *p*HBA preference. The W201Y mutant increased PCAH and PHBH activities by 4- to 21-fold. Kinetic findings showed that the W201Y mutation increased *k*_cat_ and *k*_red_ values for *p*HBA by 17-and 54-fold (Table 2). Reductions in the increase for PCA indicated that the W201Y mutation contributes less than *p*HBA to recognizing PCA. These results agreed with Tyr201 in PaPobA playing roles in hydrogen bonding with and removing protons from the 4-hydroxy group of *p*HBA (11).

### Hydrophobic cavity shape is complementary to PCA

**Figure 7:**
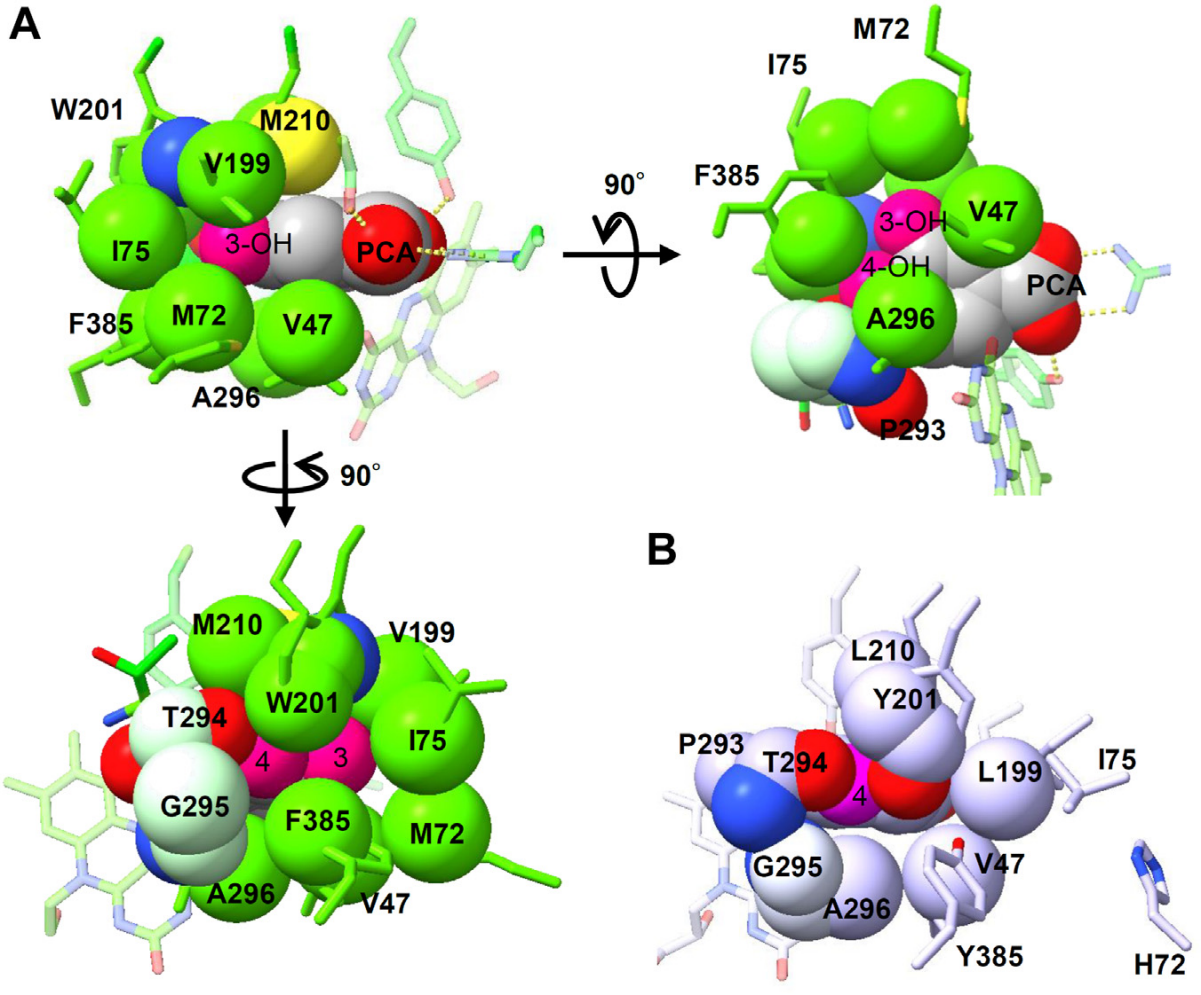
**Substrate binding at the hydrophobic cavity of XaPobA.***A*, substrate-binding site of XaPobA + PCA in CPK models rotated 90°. *B*, substrate-binding site of PaPobA + *p*HBA (PDB ID: 1IUW) in CPK model at same angle as XaPobA + PCA structure at *bottom left*.

**Figure 8:**
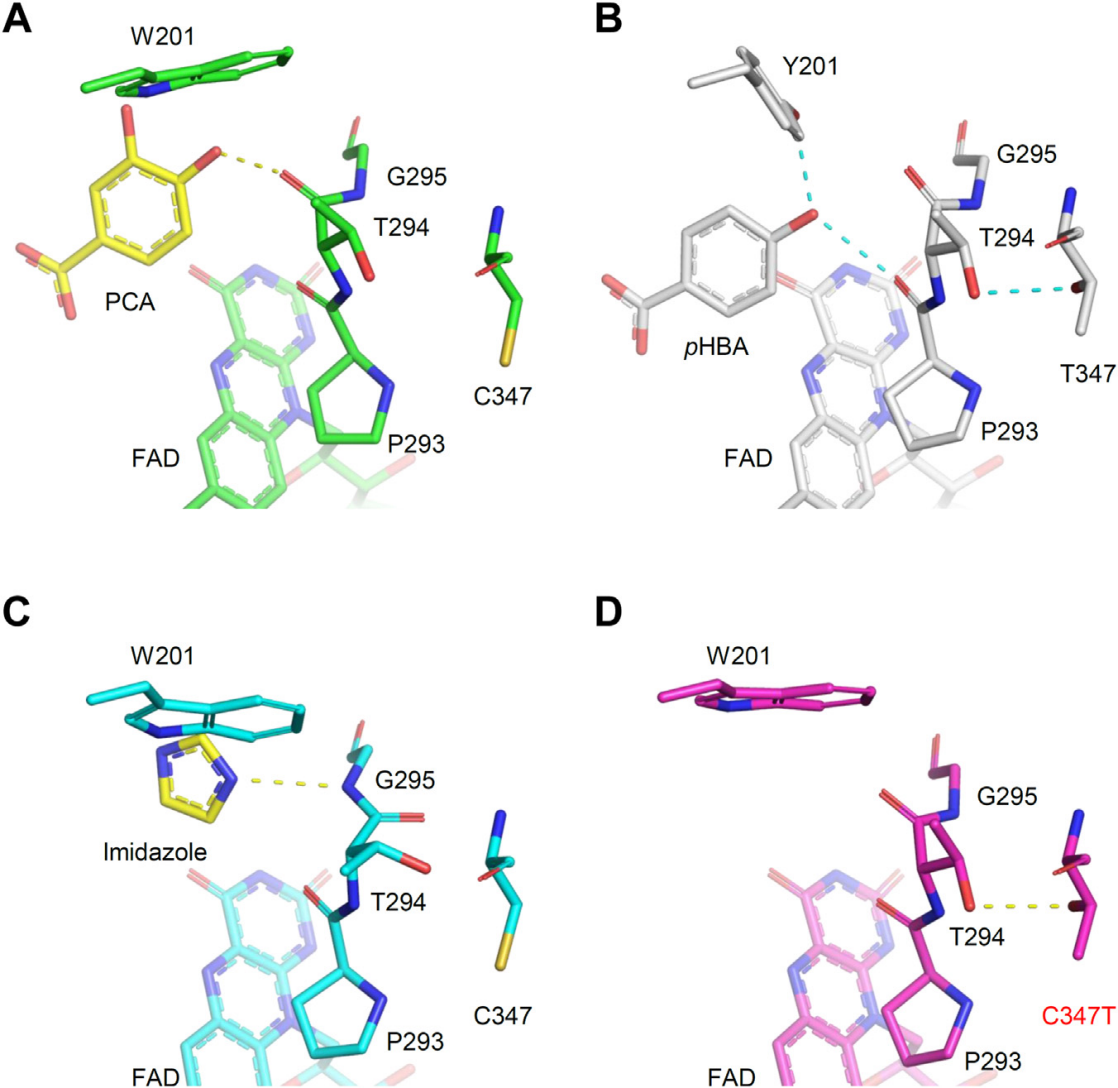
**Comparison of Thr294-Gly295 region and residue 347 in XaPobA and PaPobA.** Active sites of (*A*) XaPobA + PCA, (*B*) PaPobA complexed with *p*HBA (PDB ID: 1IUW), (*C*) XaPobA + imidazole, and (*D*) XaPobA C347T mutant. *Yellow* and *cyan dashed lines*, hydrogen bonds in XaPobA and PaPobA structures, respectively.

The Corey-Pauling-Koltun (CPK) model of the XaPobA + PCA structure showed that the side chains of eight amino acid residues formed a hydrophobic cavity with the aromatic ring of PCA. This was buried with the 3-hydroxy group of PCA enclosed by the Met72, Ile75, Val199, and Trp201 residues and faced the bottom of the cavity (Fig. 7*A*). The 4-hydroxy group of PCA was anchored through Trp201 cooperating with Phe385 and the main chain of Pro293–Gly295. The carbonyl group in the main chain of Thr294 formed a hydrogen bond with the 4-hydroxyl group of PCA and was the only hydrophilic interaction in the cavity (Fig. 8*A*). The side chains of Val47 and Ala296 formed a cavity wall parallel to the aromatic ring of PCA (Fig. 7*A*), and it was sandwiched between another cavity wall formed by Met210 at the opposite plane of the aromatic ring. We considered that these residues shaped the cavity to complement the 3,4-diphenolic group of PCA, and bind PCA at a productive orientation. This agreed with their importance in catalysis (Fig. 4*B*, Table 2). Such a hydrophobic cavity was not apparent in PaPobA; Val47 and Ala296 residues were common to XaPobA and formed a similar wall. However, the bottom-building residues were substituted with more hydrophilic Tyr201 and Tyr385, and the side chains of Tyr385 and His72 were directed away from the bound *p*HBA to establish an incomplete hydrophobic cavity (Fig. 7*B*).

The δ-sulfur atom of Met210 was close to PCA at 3.6 Å from the center of the benzene ring (Fig. 7*A* right panel). This distance was within the range (∼5 Å) for the non-covalent sulfur π-interaction proposed by bioinformatic findings and quantum mechanical calculations that have revealed a stable interaction between the sulfur atom and the aromatic ring (25). Substituting Met210 with alanine substantially impaired FAD reduction by XaPobA (Table 2) and with leucine decreased the enzyme activity to 42% of the WT (Fig. 4*B*). This indicated that the Met210 residue drives sulfur-π and hydrophobic interactions, to stabilize the XaPobA-PCA complex. This notion was consistent with methionine-aromatic interactions stabilizing the protein structure and ligand-protein interactions at 1 to 1.5 kcal mol^−1^ (25). These findings of the hydrophobic cavity and the Met210 π-interaction comprised the unique mechanism through which XaPobA can distinguish and bind PCA. This mechanism might characterize clade F PobAs, as most of them have these residues.

### Involvement of the main chain of Thr294-Gly295 for PCA binding

The hydrogen bond between the carbonyl oxygen of Thr294 and the 4-hydroxy group of PCA at a distance of 2.3 Å stabilized PCA at a catalytically active configuration (Fig. 8*A*). The corresponding Thr294 residue and *p*HBA were more distantly located in PaPobA; instead, the main chain carbonyl oxygen between Pro293 and Thr294 formed a hydrogen bond with *p*HBA (Fig. 8*B*). The side chain of Thr294 further formed a hydrogen bond with the side chain of Thr347, which might limit the conformational flexibility of a loop including Pro293-Gly295 (17). In contrast, XaPobA replaced the Thr347 residue with Cys347, which has a side chain that does not interact with Thr294. This substitution shifted the Thr294-Gly295 region towards PCA in XaPobA. The main chain peptide bond of Thr294-Gly295 was notably flipped in the XaPobA + imidazole structure (Figs. 8*C* and S10, *A* and *B*), suggesting that the Thr294‒Gly295 region was flexible due to the absence of hydrogen bond between this region and Cys347, unlike PaPobA. The *k*_cat_ value for PCA was 20-fold lower for the XaPobA C347T mutant that mimicked PaPobA, and 2-fold higher for *p*HBA, compared with WT XaPobA (Table 2). Pre-steady state FAD reduction by the C347T mutant in the presence of PCA proceeded at a 30-fold lower rate (*k*_red_ = 6.5 ± 0.7 × 10^−1^ s^−1^) than WT XaPobA. These results indicated that Cys347 is involved in the ability of XaPobA to discriminate PCA. We also determined the ligand-free crystal structure of the C347T mutant at a resolution of 2.06 Å (Table S2). The Thr347 residue formed a hydrogen bond with Thr294 and fixed the peptide bond in a conformation similar to those of XaPobA + PCA and PaPobA (Figs. 8*D* and S10*C*). Taken together, these results indicated that Cys/Thr347, located distal to the active center, plays a role in fine-tuning the position of the Thr294-Gly295 region that fits PCA to the hydrophobic cavity, and regulates the substrate preference of PobA.

## Discussion

The diversity of PobA proteins has never been fully revealed despite extensive studies over several decades. Here, we analyzed the phylogenetic relationship of 8127 amino acid sequences related to PobA. We found eight groups of PobAs, among which we identified new clades comprising biochemically uncharacterized proteins. Among them, XaPobA, OtPobA, and PkPobA belonged to clade F and were the first examples of natural PCAHs. The clade F PobAs are encoded by a few *Burkholderiales* species, whereas most bacteria in this order encode other extra PobA-like isozymes and belong to clades C or D (6). This implied that the proteins in clade F evolved separately from their bacterial lineage. Some *Burkholderiales* bacteria are likely to acquire the ancestral gene of clade F PobAs by horizontal gene transfer, which then evolved PCAHs. The NAD(P)H preference of PobAs is associated with a bacterial phylogeny (6). The amino acid residues 32‒42 that comprise helix H2 determine the NADPH preference of PaPobA (6, 26), were also found in XaPobA (Fig. S6) and are likely to be responsible for the NADPH preference of XaPobA. The physiological role of bacterial PCAH remains unknown, but the powerful antioxidant activity of the reaction product, GA, implies that PCAH protects *Burkholderiales* bacteria from oxidants in an aerobic milieu.

This study characterized the structural and biochemical properties of XaPobA and identified a hydrophobic cavity configured to fit PCA along with Met-aromatic interaction, which is a novel mechanism of PobA family proteins for substrate recognition (Fig. 7*A*). This mechanism has been adopted by XaPobA to recognize PCA during turnover because the cavity-forming hydrophobic amino acids are critical for the reaction. Shape complementarity is a proposed mechanism for protein/protein, antigen-antibody, and ligand/receptor interactions (27, 28), whereas our results implied a different mechanism that participates in enzyme-substrate interactions. The indole monooxygenase VpIndA forms a hydrophobic cavity with a good complementary fit to the substrate indole at the active site (29), but a Met-aromatic interaction is not involved. Some clade F PobAs with an unknown substrate substitute for Val199 that configures the bottom of the cavity with isoleucine, suggesting that the cavity shape for potential substrates could be diverse within clade F PobAs. Notably, these substrate recognition mechanisms were unique to XaPobA and did not participate in PaPobA, which is equipped with a relatively hydrophilic system that relays protons from the 4-hydroxy group of *p*HBA at the active center to the solvent *via* Tyr385 and Tyr201 (Fig. S1*A*) (5).

The non-covalent Met-aromatic interaction is considered to affect PCA binding in addition to simple hydrophobic interaction. The distance of 3.6 Å between the Met210 δ-sulfur atom and the ring center of PCA agreed with the prediction that Met-aromatic interaction is stronger than simple hydrophobic interactions within ∼5 Å (25). The δ-sulfur located at 13° relative to the normal orientation of the aromatic ring matches a preference of 30°–60° for Met-aromatic interaction (25). The crystal structure of XaPobA indicated that the δ-sulfur atom faces downwards toward the aromatic ring, which has been described as the down conformation (30). The computational model showed that a down conformation is energetically less favorable than an up conformation with the sulfur atom facing upwards, while the interaction contributes to an additional ∼1.5 kcal mol^−1^ (25) and facilitates stable PCA binding. Met-aromatic interactions are found in either interprotein methionine and aromatic amino acids, or protein-protein complex interfaces, and are associated with folded protein stabilization and protein functions (25, 31). Our finding of a Met-aromatic interaction between an enzyme and a substrate is novel as far as we can ascertain. The Met210 residue was conserved in clade F PobA proteins, suggesting that this interaction is common among clade F PobAs.

The absence of the Tyr201 and Tyr385 residues that are conserved among conventional PobAs is another notable feature of XaPobA. The 4-hydroxy group of *p*HBA in PaPobA is deprotonated through a hydrogen bond network established from their side chains; this is a driving force that moves FAD to the external position and results in FAD reduction by NADPH (10). Therefore, the fact that XaPobA replaces Tyr201 with Trp201 lacking a side chain atom that deprotonates under physiological conditions is notable. Nevertheless, XaPobA requires PCA to reduce FAD. One possible explanation is that the main chain carbonyl group of Thr294 deprotonates PCA. In this scenario, the protonated carbonyl of Thr294 re-protonates the 4-phenolate group of PCA after FAD reduction because a proton relay from PCA to the solvent is not obvious in XaPobA structures. Another explanation for the PCA requirement could be that XaPobA does not need its deprotonation to move FAD to the outer position, or a protein base undergoes a conformational change that deprotonates the 4-hydroxy group in the vicinity of PCA. They propose that activation of FAD might proceed without a hydrogen-bonding network or a catalytic base for substrate deprotonation at catalytic sites in phenol hydroxylase (32, 33) and the aromatic monooxygenases PgaE and CabE (34). However, the functional relevance between these enzymes and XaPobA in substrate-dependent FAD reduction awaits further investigation.

Gallic acid is a popular raw material for producing food additives and chemical reagents for microfabrication (15, 16). The present GA production process involves the chemical hydrolysis of plant-derived tannin and discharges strongly alkaline or acidic wastewater. Thus, the microbial production of GA should avoid this hydrolysis and minimize environmental load (15, 16). The PaPobA mutants that hydroxylate PCA to GA are essential for developing a microbial GA production process (17, 18, 19). Our finding of natural PCA hydroxylases and a novel mechanism of PCA recognition could expand engineered PobAs that could develop into an industrial GA production process. Our findings also revealed that the PobAs analyzed to date represent only a small fraction of diverse groups of PobAs (Fig. S2). These groups might include new PCAHs, which could lead to the discovery of PobA-related enzymes with novel catalytic properties and reaction mechanisms.

## Experimental procedures

### Strains and media

Plasmids were constructed and recombinant proteins were produced by incubating *Escherichia coli* NEB turbo (New England Biolabs, Ipswich, MA, USA) and BL21 Star (DE3) (Invitrogen, Carlsbad, CA, USA) in Luria Bertani (LB) medium (10 g L^−1^ tryptone, 5 g L^−1^ yeast extract, and 10 g L^−1^ NaCl) and Terrific broth (TB) medium (12 g L^−1^ tryptone, 24 g L^−1^ yeast extract, 13 g L^−1^ K_2_HPO_4_, 2.3 g L^−1^ KH_2_PO_4_, and 5 g L^−1^ glycerol), respectively. Transformants were cultured in these media supplemented with 30 mg L^−1^ kanamycin sulfate.

### Multiple sequence alignment and phylogenetic analysis

Amino acid sequences of proteins annotated as 4-hydroxybenzoate 3-monooxygenase were downloaded from the UniProtKB/TrEMBL databases. We analyzed the phylogenetics of 8127 amino acid sequences comprising 201‒600 residues using MAFFT version 7 (35) and FastTree 2.1 (36). Branch lengths of <0.5 in the tree were collapsed. Proteins in clade F and 33 close relatives in the NCBI with amino acid sequence similarity >60%) were combined, and redundant sequences were eliminated. The resulting 37 amino acid sequences were aligned and their phylogeny was analyzed using ClustalW (37) and the maximum likelihood method. Numbers along with branches indicate values calculated from 1000 bootstrap resampling replicates. The amino acid sequences of XaPobA, OtPobA, PkPobA, PaPobA, AbPobA, and CgPobA were aligned using ClustalW and visualized using ESPript (38).

### Plasmid construction

The codons of *xapobA*, *otpobA,* and *pkpobA* were optimized for *E. coli*, and then corresponding DNA fragments were synthesized. We amplified DNA by PCR using these fragments as templates with primers (Table S3), which were cloned into modified pET28a (39) digested beforehand with *Nde*Ⅰ and *Hin*dIII using NEBuilder HiFi DNA Assembly Master Mix (New England Biolabs, Ipswich, MA, USA) to yield pET28a-*xapobA*, pET28a-*otpobA*, and pET28a-*pkpobA*. A DNA fragment for *papobA* was amplified using the total DNA of *P. aeruginosa* JCM 14847 as a template with primers (Table S3) to generate pET28a-*papobA* as described above. Expression vectors for producing XaPobA mutants were constructed as follows. Vectors with a mutation in a cloned gene were amplified by PCR using primers (Table S3) and pET28a-*xapobA* as a template. The resulting DNA fragments were digested with *Dpn*I, and incubated with T4 polynucleotide kinase (Takara Bio Inc, Shiga, Japan) and Ligation high Ver. 2 (Toyobo, Kyoto, Japan) at 16 °C to generate expression vectors.

### Preparation of recombinant PobA proteins

The expression vectors were transformed into *E. coli* BL21 Star (DE3) which was cultured overnight in LB medium (5 ml) containing 30 mg l^−1^ kanamycin sulfate at 37 °C. Portions of these cultures (1 ml) were inoculated into TB medium (100 ml) containing 30 mg l^−1^ kanamycin sulfate and incubated at 37 °C with agitation at 120 rpm. Isopropyl thiogalactopyranoside (0.4 mM) was added to the medium when the optical density at 600 nm of this culture reached 0.6, then the cells were further incubated for 12 h at 20 °C with agitation at 120 rpm. The cells were collected by centrifugation, suspended in buffer A (20 mM Tris-HCl, pH 7.9), disrupted by ultrasonication, and centrifuged at 10,000*g* for 15 min. Cell debris was removed, and then proteins were purified using HisTrap FF columns (Cytiva, Marlborough) that were equilibrated with buffer A containing 20 mM imidazole. Proteins bound to resin were washed with 10-column volumes of buffer A containing 20 mM imidazole, then eluted with buffer A containing 300 mM imidazole. The eluents were concentrated and the buffer was replaced with buffer A using Amicon Ultra-0.5 ml filter devices (10 kDa cutoff) (Merck). Protein concentrations were determined using Protein Assay Dye Reagent (Bio-Rad Laboratories Inc). Purified preparations were boiled and centrifuged at 10,000*g* then FAD and FMN were analyzed. The supernatant was analyzed using an Agilent 1260 Infinity HPLC instrument (Agilent Technologies) and Purospher Star RP-18 end-capped columns (Millipore-Merck,). The mobile phase comprised a ratio of 60:40 solvent A/solvent B (solvent A: 20 mM phosphoric acid, pH 2.5; solvent B, methanol). The flow rate was 1.0 ml min^−1^ and absorption was monitored at 450 nm. The column temperature was 30 °C.

### Steady state kinetics of NAD(P)H oxidation

We purchased the following reagents from the respective suppliers: *p*HBA and GA (Wako Pure Chemical Industries (Osaka, Japan), PCA (Tokyo Chemical Industries, Tokyo, Japan), NADPH, and NADH (Oriental Yeast, Tokyo, Japan). Reaction mixtures (typically 100 μl) contained 20 mM Tris-HCl (pH 7.9), 0.25 mM NAD(P)H, 0‒2 mM PCA or *p*HBA, and purified PobAs. Adding PCA or *p*HBA started the reaction, then the absorbance of NADPH at 340 nm was monitored at 25 °C using a U3900 spectrophotometer (Hitachi, Tokyo, Japan). The initial velocity of NADPH consumption was determined in triplicate, and fitted to the Michaelis−Menten equation according to the nonlinear regression to calculate apparent Michaelis-Menten (*K*_m_) and kinetic (*k*_cat_) constants. The molar coefficient of NAD(P)H was 6300 M^−1^ cm^−1^.

### Determination of substrate oxidation rate

We incubated 100 μl of 20 mM Tris-HCl (pH 7.9) containing 0.5 mM NADPH, 2 mM *p*HBA or PCA, and purified PobA (10 μg) at 30 °C for 30 min. We then added 100 μl of 2 M HCl, and analyzed substrate oxidation the reaction by HPLC using the Agilent 1260 Infinity instrument. The initial mobile phase consisted of 90:10 solvent A/solvent B, which was gradually changed to 40:60 over 8 min and maintained at this ratio for 4 min. The flow rate was 0.8 ml min^−1^ and absorption at 280 nm was monitored. The column temperature was 30 °C.

### Pre-steady state reaction

An RSP-2000, stopped-flow rapid scan spectrometer with a photodiode-array detector (Unisoku, Osaka, Japan) was conditioned three to five times with a nitrogen gas-purged solution of 20 mM Tris-HCl (pH 7.9), 2 mM D-glucose, 10 units mL^−1^ glucose oxidase, and 150 units mL^−1^ catalase, then pre-steady state FAD reduction was measured. Nitrogen gas was flushed into reservoirs to replace headspace air. We rapidly mixed PCA or *p*HBA (2 mM) and XaPobA or its mutants in 20 mM Tris-HCl (pH 7.9) containing excess NADPH to start the reaction, then 5000 absorption spectra were collected at 25 °C. Reactions contain 2 mM D-glucose, 10 units mL^−1^ glucose oxidase, and 150 units mL^−1^ catalase. Changes in absorbance at 440 nm were averaged (*n* = 3) and fitted to single exponential functions to determine the apparent rate constants (*k*_red_) for FAD reduction.

### X-ray crystallography

We purified XaPobA and XaPobA C347T mutants (with 6× His each) as described above. Crystals of XaPobA + imidazole, PCA, and C347T were then grown at 20 °C using sitting drop vapor diffusion with a mixture of 5‒10 mg mL^−1^ protein in 0.5 μl of 20 mM Tris-HCl (pH 8.0) and equal volumes of reservoir solutions. These solutions comprised 0.24 M calcium acetate, 8% (w/v) PEG8000 (pH 8.0), and 0.1 M HCl (pH 8.0), with or without imidazole to crystallize XaPobA + imidazole and imidazole-free crystals, respectively. The reservoir solution was supplemented with 50 mM PCA for soaking. The reservoir solution was 0.24 M calcium acetate, 0.1 M imidazole-HCl (pH 8.0), and 10% (w/v) PEG8000 (pH 8.0) to crystallize the XaPobA C347T mutant. The crystals were cryoprotected in a reservoir solution supplemented with 20% (w/v) glycerol and flash-cooled by dipping them into liquid nitrogen to collect data.

### Data collection, structure determination, and refinement

X-ray diffraction data were collected at 100 K on the beamlines of SPring-8 and the Photon Factory (High Energy Accelerator Research Organization [KEK], Tsukuba, Japan). Diffraction images were processed using XDS (40). The initial structure was solved by molecular replacement using MOLREP (41), and the structure of PaPobA (PDB ID: 1IUW, sequence identity, 47.0%) was the search model. We manually rebuilt and refined models using Coot (42), REFMAC5 (43), and PHENIX (44). Occupancies of all four PCA molecules in the crystal structure were set to 1.0, and their B-factors were comparable to the protein atoms after refinement (Table S2). Molecular graphic images were prepared using PyMOL (Schrödinger, LLC) and UCSF ChimeraX 1.4 (45).

### Statistical analysis

All data are presented as means ± standard deviation. Paired groups were compared using unpaired Student’s *t*-tests.

## Data availability

The nucleotide sequences of the codon-optimized *xapobA*, *otpobA*, and *pkpobA* genes have been deposited into the Genbank database with accession numbers OR631772, OR631773, and OR631774, respectively. Atomic coordinates and structure factors (codes 8JQO, 8JQP, and 8JQQ) have been deposited into the Protein Data Bank (http://wwpdb.org/). All other data are contained in the article and supporting information.

## Supporting information

This article contains supporting information.

## Conflict of interest

The authors declare that they have no conflict of interest with the contents of this article.
