# Supplementary material for: Protocatechuate hydroxylase is a novel group A flavoprotein monooxygenase with a unique substrate recognition mechanism
Source: J Biol Chem. 2023 Nov 28;300(1):105508. doi: 10.1016/j.jbc.2023.105508 (PMC10770758; doi:10.1016/j.jbc.2023.105508)
Supplement: Supporting Figures S1–S10 and Tables S1–S3 [file mmc1.docx]

**Supporting information**

Protocatechuate hydroxylase is a novel group A flavoprotein monooxygenase with a unique substrate recognition mechanism

**Nozomi Katsuki^1,†^, Riku Fukushima^2,†^, Yuki Doi^1^, Shunsuke Masuo^1^, Takatoshi Arakawa^3^, Chihaya Yamada^4^, Shinya Fushinobu^2,5,*^, and Naoki Takaya^1,*^**

From ^1^Faculty of Life and Environmental Sciences, Microbiology Research Center for Sustainability, University of Tsukuba, 1-1-1 Tennodai, Tsukuba, Ibaraki 305-8572, Japan; ^2^Department of Biotechnology, The University of Tokyo, Tokyo, 113-8657, Japan; ^3^Faculty of Pharmaceutical Sciences, Tokyo University of Science, Noda, Chiba 278-8510, Japan; ^4^School of Agriculture, Meiji University, Kawasaki, Kanagawa 214-8571, Japan; ^5^Collaborative Research Institute for Innovative Microbiology, The University of Tokyo, Tokyo, 113-8657, Japan

**Supplementary Figure S1.** Structure of PaPobA active site.

**Supplementary Figure S2.** Phylogenetic tree of PobA-related proteins.

**Supplementary Figure S3.** Biochemical characterization of cofactors.

**Supplementary Figure S4.** Analyses of reaction products of XaPobA, OtPobA, and PkPobA.

**Supplementary Figure S5.** Steady-state kinetics of XaPobA, OtPobA, and PkPobA.

**Supplementary Figure S6.** Alignment of PobA protein sequences.

**Supplementary Figure S7.** Dimer structure and electron density maps of ligands.

**Supplementary Figure S8.** Inner and outer conformations of FAD and ligand binding sites in PobA proteins.

**Supplementary Figure S9.** Two binding modes of PCA in *Pseudomonas* PobAs.

**Supplementary Figure S10.** Electron density maps of Cys/Thr347 and Thr294-Gly295.

**Supplementary Table S1.** Specific activities of XaPobA, OtPobA, and PkPobA.

**Supplementary Table S2.** Data collection and refinement statistics.

**Supplementary Table S3.** Primers for PCR amplification.

**Supplementary Figure S1.** Structure of PaPobA active site.

(A, B) Views from different directions mainly of *p*HBA complex structure of PaPobA (PDB ID: 1IUW). *Superimposed bridging water and FAD in outer conformation of different crystal structures (PDB IDs: 1IUT and 1DOD, respectively).

**
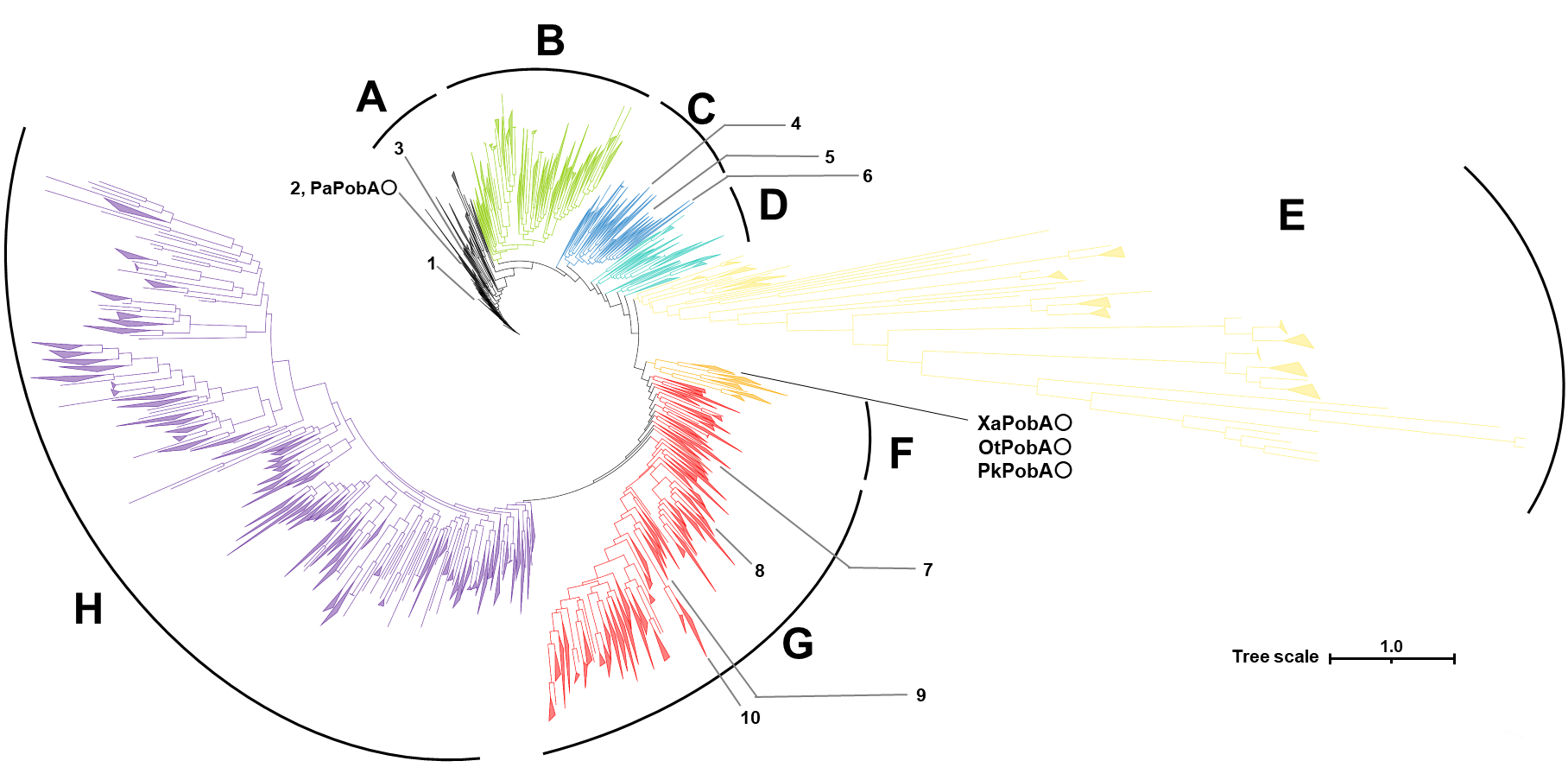
**

**Supplementary Figure S2.** Phylogenetic tree of PobA-related proteins.

Amino acid sequences of 8,127 PobA-related proteins downloaded from UniProtKB/TrEMBL databases were aligned using MAFFT ver. 7, processed by FastTree 2.1, then phylogenetic tree was constructed using iTOL. Leaves with average branch length of < 0.5 in the tree were collapsed into a single clade. The proteins were grouped into eight clades A-H. XaPobA, OtPobA, PkPobA, and previously characterized proteins are highlighted. Numbers indicate; 1, Q9F1J8, Q06519; 2, P20586, V6A7D3; 3, Q9X7I6, O30873; 4, Q59744; 5, Q46R66; 6, Q03298: 7, Q46U22; 8, C4TP09; 9, A0A0U1URQ7, A0A8E3PMG9: 10, Q8NRH2.

**
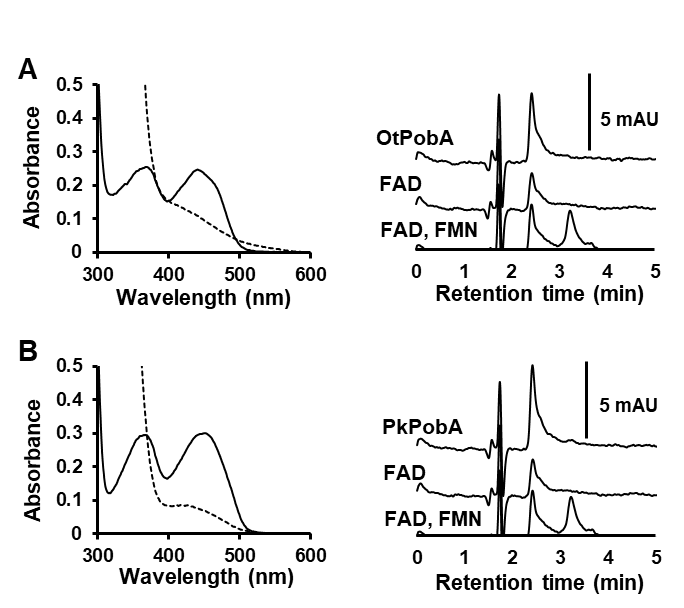
**

**Supplementary Figure S3.** Biochemical characterization of cofactors.

(A) Left panel, Absorption spectra of OtPobA (46 µM) in 20 mM Tris-HCl (pH 7.9). Black line, oxidized enzyme. Dashed line, enzyme reduced by 5 mM sodium hydrosulfite. Right panel, analysis of cofactor in OtPobA by HPLC. (B) These were analyzed as described in (A) using PkPobA (46 µM).

　　　　　
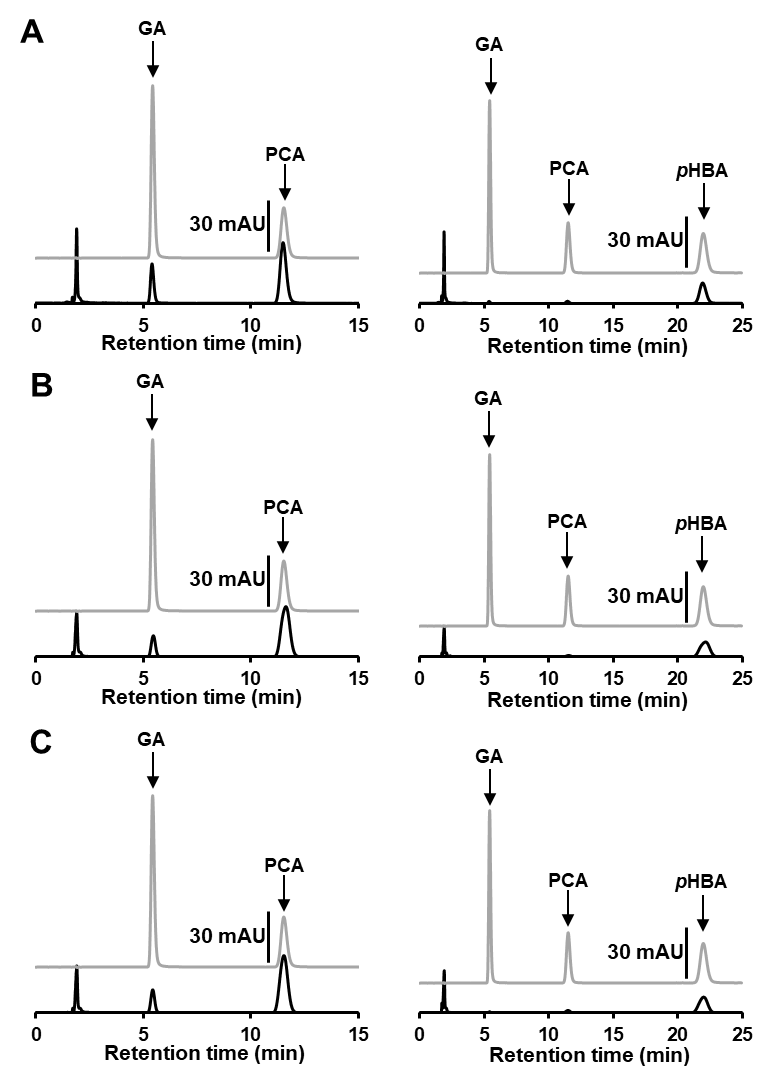


**Supplementary** **Figure S4.** Analyses of reaction products of XaPobA, OtPobA, and PkPobA.

(A) HPLC analyses of mixtures containing 10 µg XaPobA, 0.5 mM NADPH, and aromatic substrates 2 mM PCA (left panel) and 1 mM *p*HBA (right panel) reacted in 20 mM Tris-HCl (pH 7.9) for 30 min at 30℃. Black and gray traces, reaction mixture and standards (*p*HBA, PCA, and GA 10 mg L^-1^ each), respectively. The analyses included 10 µg each of OtPobA (B) and PkPobA (C) and proceeded as described in (A).

　
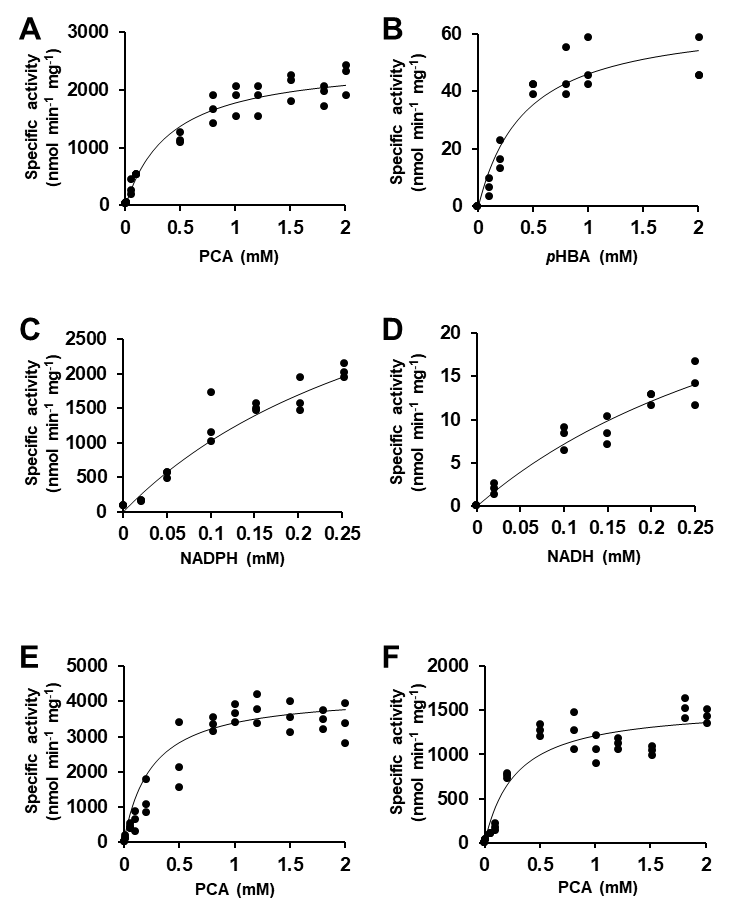


**Supplementary** **Figure S5.** Steady-state kinetics of XaPobA, OtPobA, and PkPobA.

Initial velocity of PCA/*p*HBA-dependent NAD(P)H oxidation was determined to calculate *K*_m_ and *k*_cat_ values of PobAs. (A ‒D) Analyses of XaPobA proceeded in reaction mix containing 0‒2 mM PCA (A) or *p*HBA (B) and 0.25 mM NADPH, 0‒0.25 mM NADPH (C) or NADH (D) and 2 mM PCA. We analyzed (E) OtPobA and (F) PkPobA in reaction mixes containing 0‒2 mM PCA and 0.25 mM NADPH. Experimental data in triplicate were fitted to the Michaelis-Menten equation.


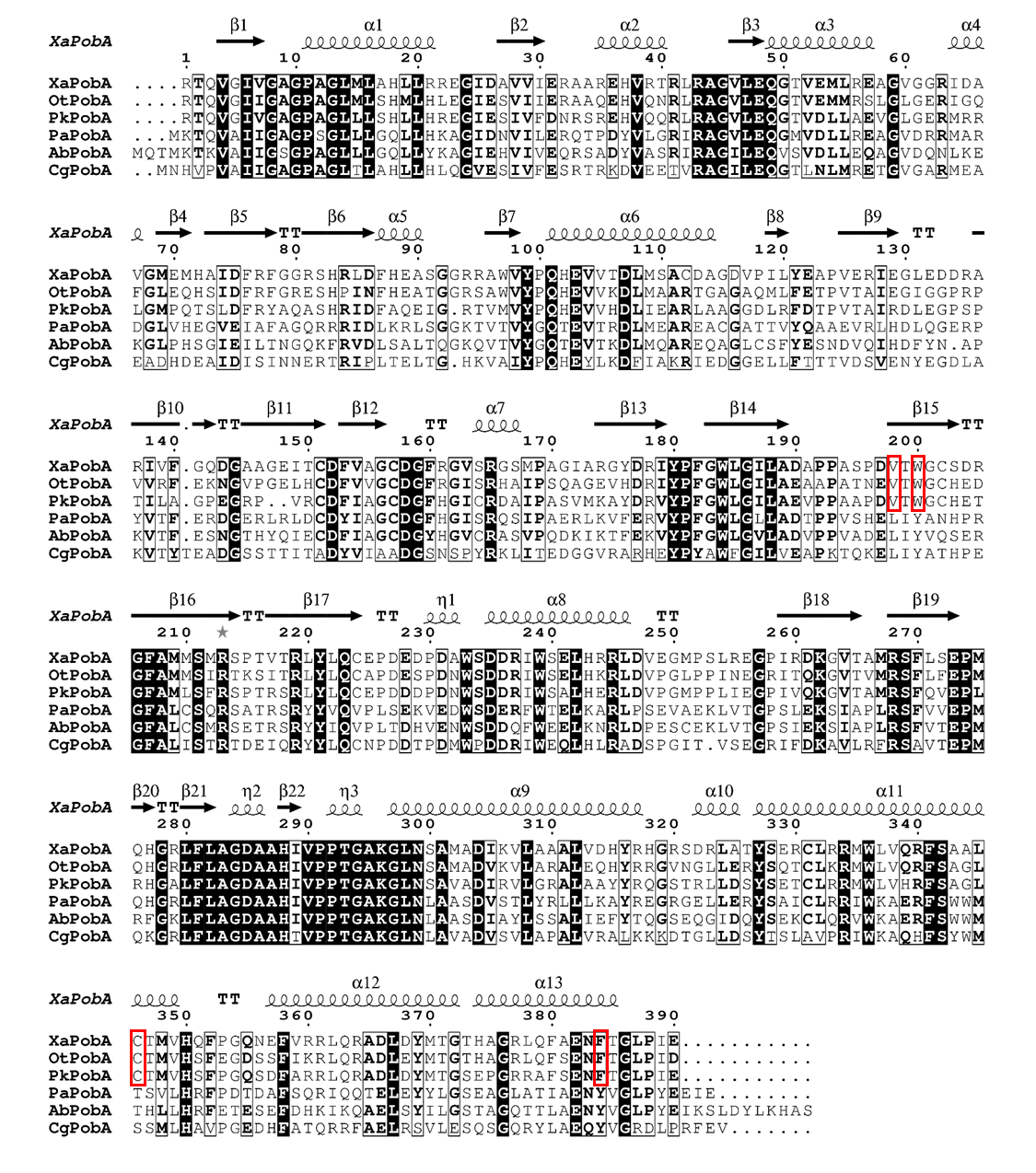


**Supplementary Figure S6.** Alignment of PobA protein sequences.

Amino acid sequences of XaPobA, OtPobA, PkPobA, PaPobA (UniProt ID: P20586, clade A), AbPobA (PobA from *Acinetobacter baylyi*, UniProt ID: Q03298, clade C), and CgPobA (PobA from *Corynebacterium glutamicum*, UniProt ID: Q8NRH2, clade G) were aligned using ClustalW, and the resulting figure was created using ESPript. Secondary structures assigned to XaPobA (PDB ID: 8JQP) are indicated above sequences. Black boxes, identical residues. Bold letters, similar amino acids. Red boxes, unique residues for clade F PobAs.

**Supplementary Figure S7.** Dimer structure and electron density maps of ligands.

(A) Dimer structures of XaPobA + imidazole (left, cyan) and XaPobA + PCA (right, green). Dimer counterpart chains (white ribbons), FAD (white sticks), and imidazole and PCA (yellow sticks). Polder maps of (B) imidazole in the active site of XaPobA + imidazole (4.0σ) and (C) PCA in the active (upper panel) and second (lower panel) sites of XaPobA + PCA (4.5σ).

**
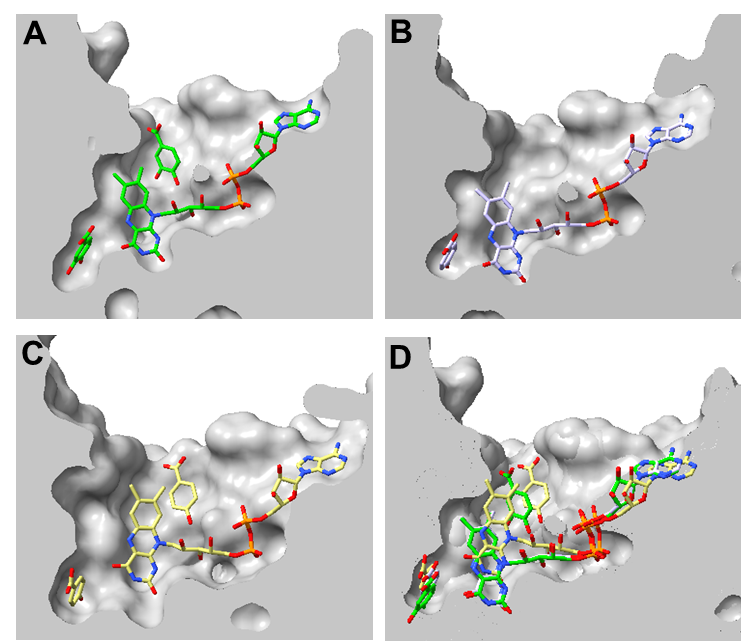
**

**Supplementary** **Figure S8.** Inner and outer conformations of FAD and ligand binding sites in PobA proteins.

(A) Inner conformation of FAD and two PCA molecules in XaPobA + PCA (PDB ID: 8JQP). (B) Inner conformation of FAD and *p*HBA in PaPobA WT (PDB ID: 1IUW). (C) Outer conformation of FAD and two *p*HBA molecules in PaPobA R220Q mutant (PDB ID: 1K0I). (D) Superposition of (A) (green), (B) (white), and (C) (yellow).

**Supplementary Figure S9.** Two binding modes of PCA in *Pseudomonas* PobAs.

(A) Productive binding mode of Y385F mutant in PaPobA, which hydroxylates PCA (PDB ID: 6JU1). (B) Inert binding orientation of WT PfPobA, with low PCA hydroxylation activity (PDB ID: 1PHH).


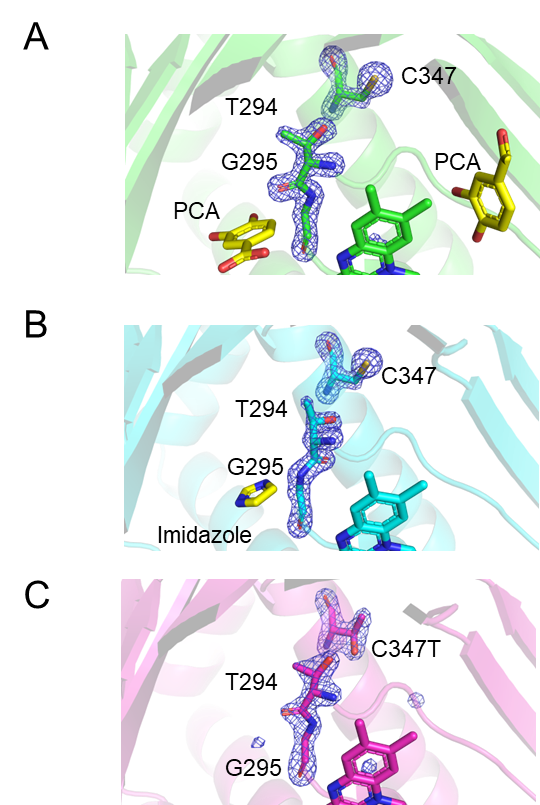


**Supplementary Figure S10.** Electron density maps of Cys/Thr347 and Thr294-Gly295.

Polder maps show (A) Cys347 and Thr294-Gly295 in WT XaPobA + PCA (6.0σ) (PDB ID: 8JQP). (B) Cys347 and Thr294-Gly295 in WT XaPobA + imidazole (6.0σ) (PDB ID: 8JQO). (C) Thr347 and Thr294-Gly295 in XaPobA C347T mutant (6.0σ) (PDB ID: 8JQQ).

**Supplementary Table S1.** Specific activities of XaPobA, OtPobA, and PkPobA

| Enzymes | NAD(P)H | Specific activity (nmol min^-1^ mg^-1^)* | |
| --- | --- | --- | --- |
|  |  | PCA**^†^** | *p*HBA |
| XaPobA | NADPH  NADH | 1.8 ± 0.2 × 10^3^  1.3 ± 0.8 × 10^1^ | 4.8 ± 0.7 × 10^1^  9.7 ± 2.6 |
| OtPobA | NADPH  NADH | 2.7 ± 0.2 × 10^3^  2.3 ± 0.5 × 10^1^ | 8.7 ± 1.8 × 10^2^  < 1.0 |
| PkPobA | NADPH  NADH | 1.7 ± 0.3 × 10^3^  3.4 ± 0.2 × 10^1^ | 1.1 ± 0.4 × 10^2^  < 1.0 |

*Rates of aromatic substrate-dependent NAD(P)H oxidation measured using 1 mM PCA/*p*HBA and 0.25 mM NAD(P)H. Data are shown as means ± standard deviation (*n* = 3). **^†^**Activities of 1 mM each of *p*-aminobenzoate, hydroquinone, catechol, *o*-hydroxybenzoate, *m*-hydroxybenzoate, 2,4-dihydroxybenzoate, *p*-coumarate, and caffeate were below limits of detection.

**Supplementary Table S2.** Crystallographic data collection and refinement statistics

|  | XaPobA + imidazole | XaPobA + PCA | XaPobA C347T |
| --- | --- | --- | --- |
| Data collection^*^ |  |  |  |
| Beamline | SPring-8 BL45XU | KFK-PF BL-5A | KFK-PF BL-5A |
| Space group | *P*2_1_ | *P*2_1_ | *P*2_1_2_1_2_1_ |
| Unit cell (Å, °) | *a* =88.260, *b* =67.389, *c* =128.234, *β* =90.672 | *a* =72.966, *b* =65.074, *c* =80.681, *β* =107.546 | *a* =66.961, *b* =87.816, *c* =128.617 |
| Resolution (Å) | 46.45–1.60 | 47.52-1.65 | 49.20-2.06 |
| Total reflections | 669,317 (31,391) | 291,661 (14,204) | 315,011 (24,350) |
| Unique reflections | 194,834 (9,626) | 86,275 (4,251) | 47,447 (3,637) |
| *R*_merge_ | 0.066 (0.500) | 0.048 (0.497) | 0.084 (0.648) |
| *R*_pim_ | 0.039 (0.311) | 0.031 (0.319) | 0.035 (0.271) |
| Mean *I*/*σ*(*I*) | 10.6 (2.4) | 13.3 (2.2) | 16.4 (3.2) |
| CC_1/2_ | 0.995 (0.795) | 0.999 (0.816) | 0.988 (0.883) |
| Completeness (%) | 98.8 (98.8) | 100.0 (99.9) | 100.0 (100.0) |
| Multiplicity | 3.4 (3.3) | 3.4 (3.3) | 6.6 (6.7) |
| Mol/ASU **^†^** | 4 | 2 | 2 |
| Refinement |  |  |  |
| Resolution (Å) | 46.45-1.60 | 47.52-1.65 | 46.38-2.06 |
| Reflections (n) | 194,812 | 86,210 | 47,363 |
| *R*_work_/ *R*_free_ | 0.2228/0.2465 | 0.1818/0.2212 | 0.2270/0.2577 |
| Number of atoms |  |  |  |
| Amino acids | 11,467 | 6,069 | 6,068 |
| Ions | 4 | 2 | 1 |
| Ligands | 247 | 162 | 106 |
| Waters | 685 | 601 | 139 |
| Average B-factor (Å^2^)^‡^ |  |  |  |
| Protein | 20.0/21.2/31.2/39.9 | 20.3/20.2 | 28.3/38.6 |
| FAD | 16.0/16.4/28.1/56.6 | 13.5/14.5 | 23.0/35.5 |
| Ligands | 25.2/27.2/30.5/39.2 (imidazole) | 19.8/24.8 (active site PCA)  24.0/25.9 (second site PCA)  22.0/37.2 (glycerol) |  |
| Ions | 27.3 (Ca^2+^) | 16.3 (Ca^2+^) | 30.6 (Ca^2+^) |
| Waters | 32.0 | 26.5 | 35.3 |
| RMSD from ideal values | |  |  |
| Bond lengths (Å) | 0.005 | 0.009 | 0.002 |
| Bond angles (°) | 0.773 | 1.028 | 0.433 |
| Ramachandran plot (%) | |  |  |
| Favored | 97.87 | 98.46 | 97.81 |
| Allowed | 2.13 | 1.54 | 2.19 |
| Outlier | 0.00 | 0.00 | 0.00 |
| PDB code | 8JQO | 8JQP | 8JQQ |

*Values in parentheses represent highest resolution shell. ^†^Molecules per asymmetric unit (n). ^‡^Values of the polypeptides and ligand molecules in the A/B/C/D or A/B chains are separately shown.

**Supplementary Table S3.** Primers for PCR amplification

| Primer | Nucleotide sequence (5’⭢3’) | Application |
| --- | --- | --- |
| *Construction of vectors for PobAs* | |  |
| xaF | CTGGTGCCGCGCGGCAGCCATATGCGCACGCAAGTGGGT | XaPobA |
| xaR | GGTGCTCGAGTGCGGCCGCAAGCTTATTCGATCGGCAAGCCGG |  |
| otF | CTGGTGCCGCGCGGCAGCCATATGCGCACTCAGGTCGGTATTATC | OtPobA |
| otR | GGTGCTCGAGTGCGGCCGCAAGCTTAGTCAATGGGCAGACCC |  |
| pkF | CTGGTGCCGCGCGGCAGCCATATGCGCACCCAAGTGGGTATC | PkPobA |
| pkR | GGTGCTCGAGTGCGGCCGCAAGCTTATTCGATCGGCAGGCCC |  |
| paF | CTGGTGCCGCGCGGCAGCCATATGAAGACTCAAGTCGCC | PaPobA |
| paR | GGTGCTCGAGTGCGGCCGCAAGCTTCTACTCGATTTCCTCGTAG |  |
| *Production of XaPobA mutants* | |  |
| xaW201AF | CCGGACGTTACGGCGGGCTGCTCCGATC | W201A |
| xaW201AR | GATCGGAGCAGCCCGCCGTAACGTCCGG |  |
| xaW201YF | TATGGCTGCTCCGATCGCGGCTTTG | W201Y |
| xaW201YR | CGTAACGTCCGGACTCGCTGGC |  |
| xaM210AF | GCGATGTCTATGCGTTCGCCGAC | M210A |
| xaM210AR | AGCAAAGCCGCGATCGGAGC |  |
| xaM210LF | TTGATGTCTATGCGTTCGCCGAC | M210L |
| xaM210LR | AGCAAAGCCGCGATCGGAGC |  |
| xaV199AF | GCTACGTGGGGCTGCTCCGATCG | V199A |
| xaV199AR | GTCCGGACTCGCTGGCGGTGCATC |  |
| xaV199LF | CTTACGTGGGGCTGCTCCGATCG | V199L |
| xaV199LR | GTCCGGACTCGCTGGCGGTGCATC |  |
| xaV199SF | AGTACGTGGGGCTGCTCCGATCG | V199S |
| xaV199SR | GTCCGGACTCGCTGGCGGTGCATC |  |
| xaC347TF | ACCACCATGGTCCACCAGTTTCCAG | C347T |
| xaC347TR | CAGTGCCGCGCTAAAGCGTTG |  |
| xaF385YF | TACACCGGCTTGCCGATCGAATAAG | F385Y |
| xaF385YR | GTTTTCGGCGAACTGCAGGCG |  |
| xaR214KF | AAGTCGCCGACGGTAACCCGGC | R214K |
| xaR214KR | CATAGACATCATAGCAAAGCCGCGATC |  |
| xaS212AF | GCTATGCGTTCGCCGACGGTAACCC | S212A |
| xaS212AR | CATCATAGCAAAGCCGCGATC |  |
| xaY222AF | GCTCTGCAGTGCGAACCGGATG | Y222A |
| xaY222AR | CAGCCGGGTTACCGTCGGCG |  |
